# UEV-1 Is an Ubiquitin-Conjugating Enzyme Variant That Regulates Glutamate Receptor Trafficking in *C. elegans* Neurons

**DOI:** 10.1371/journal.pone.0014291

**Published:** 2010-12-13

**Authors:** Lawrence B. Kramer, Jaegal Shim, Michelle L. Previtera, Nora R. Isack, Ming-Chih Lee, Bonnie L. Firestein, Christopher Rongo

**Affiliations:** 1 The Waksman Institute, Department of Genetics, Rutgers, The State University of New Jersey, Piscataway, New Jersey, United States of America; 2 Cancer Experimental Resources Branch, National Cancer Center, Goyang, Korea; 3 Department of Cell Biology and Neuroscience, Rutgers, The State University of New Jersey, Piscataway, New Jersey, United States of America; 4 Graduate Program in Molecular Biosciences, Rutgers, The State University of New Jersey, Piscataway, New Jersey, United States of America; Columbia University, United States of America

## Abstract

The regulation of AMPA-type glutamate receptor (AMPAR) membrane trafficking is a key mechanism by which neurons regulate synaptic strength and plasticity. AMPAR trafficking is modulated through a combination of receptor phosphorylation, ubiquitination, endocytosis, and recycling, yet the factors that mediate these processes are just beginning to be uncovered. Here we identify the ubiquitin-conjugating enzyme variant UEV-1 as a regulator of AMPAR trafficking *in vivo*. We identified mutations in *uev-1* in a genetic screen for mutants with altered trafficking of the AMPAR subunit GLR-1 in *C. elegans* interneurons. Loss of *uev-1* activity results in the accumulation of GLR-1 in elongated accretions in neuron cell bodies and along the ventral cord neurites. Mutants also have a corresponding behavioral defect—a decrease in spontaneous reversals in locomotion—consistent with diminished GLR-1 function. The localization of other synaptic proteins in *uev-1*-mutant interneurons appears normal, indicating that the GLR-1 trafficking defects are not due to gross deficiencies in synapse formation or overall protein trafficking. We provide evidence that GLR-1 accumulates at RAB-10-containing endosomes in *uev-1* mutants, and that receptors arrive at these endosomes independent of clathrin-mediated endocytosis. UEV-1 homologs in other species bind to the ubiquitin-conjugating enzyme Ubc13 to create K63-linked polyubiquitin chains on substrate proteins. We find that whereas UEV-1 can interact with *C. elegans* UBC-13, global levels of K63-linked ubiquitination throughout nematodes appear to be unaffected in *uev-1* mutants, even though UEV-1 is broadly expressed in most tissues. Nevertheless, *ubc-13* mutants are similar in phenotype to *uev-1* mutants, suggesting that the two proteins do work together to regulate GLR-1 trafficking. Our results suggest that UEV-1 could regulate a small subset of K63-linked ubiquitination events in nematodes, at least one of which is critical in regulating GLR-1 trafficking.

## Introduction

Excitatory synaptic communication in the central nervous system is mediated by the neurotransmitter glutamate and the glutamate receptor ion channels that receive and propagate glutamatergic signaling at the postsynaptic membrane [1]. Glutamatergic synapses show substantial plasticity, becoming dynamically weakened or strengthened in an activity dependent manner [2]–[6]. The trafficking of glutamate receptors, particularly AMPA-type receptors (AMPARs), in and out of the postsynaptic membrane is emerging as a key mechanism underlying synaptic and behavioral plasticity [5], [7]. Thus, robust molecular and cellular models for learning and memory will require a full understanding of the various mechanisms and molecules that regulate AMPAR trafficking.

AMPAR trafficking involves several steps, including initial delivery to the synaptic membrane, anchoring, endocytosis, and finally sorting either to the lysosomal pathway for degradation or to the synaptic membrane via various recycling pathways [8], [9]. AMPARs are tetrameric [10], and each subunit has cytoplasmic tail sequences that determine its subcellular trafficking [11]–[16]. These tail sequences direct both AMPAR endocytosis and exocytosis in response to neural activity by interacting with kinases and phosphatases, PDZ scaffolding molecules, and the endocytosis machinery [2], [17], [18].

The Ubiquitin Proteasome System (UPS) also regulates AMPAR trafficking [18]–[20]. AMPARs, like many membrane proteins, can be post-translationally modified by the addition of single ubiquitin molecules (monoubiquitination) [21]. Monoubiquitination often serves as a signal for their internalization [19], [22], [23]. In addition, polyubiquitination can occur through the addition of new ubiquitin molecules to the final ubiquitin molecule in a growing polyubiquitin chain that is initially attached to a substrate protein [24]. Typically this occurs through the formation of a covalent link between the carboxy-terminus of the incoming ubiquitin and the lysine 48 (K48) residue of the final ubiquitin in the chain. Once four or more ubiquitins are added, the polyubiquitinated protein becomes a degradation substrate for the 26S proteasome.

Polyubiquitin chains can also be formed through linkages other than K48. For example, polyubiquitin chains can be assembled through K63 linkages. K63-linked polyubiquitination is generally not thought to target substrates to the proteasome; rather, it appears to have a more regulatory role [25]. For example, K63-ubiquitination has emerged as an additional mechanism by which endocytosed proteins become shunted to Multivesicular Bodies (MVBs) for degradation [26]–[30]. One family of proteins implicated in K63-linked ubiquitination is the UEV protein family. UEV proteins share sequence similarity with ubiquitin E2 conjugating enzymes, but lack the catalytic cysteine present in functional E2 enzymes [31]–[35]. One well-studied UEV protein is the ESCRT complex subunit Vps23, which routes ubiquitinated membrane receptors from the endosome to MVBs and eventually to lysosomes for degradation [36], [37]. Other known UEV proteins form heterodimers with the E2 enzyme Ubc13 to catalyze the formation of K63-linked polyubiquitin chains [38]. UEV/Ubc13 heterodimers are implicated in several biological processes, including the immune response [39], lysosomal targeting [27], [30], [40], [41], the formation of protein inclusions [42], [43], DNA repair [44], protein transport [45], and oncogenic transformation [46]. While UEV proteins have been examined extensively in yeast and in mammalian cell culture, little is known about their function *in vivo* in metazoans. Moreover, it is unknown whether K63-linked ubiquitination plays a role in AMPAR trafficking.

The role of ubiquitination in AMPAR trafficking can be studied *in vivo* in the model organism *C*. *elegans.* The *C*. *elegans* genome contains two AMPA-type subunits: GLR-1 and GLR-2 [47]–[49]. Fluorescently tagged versions of GLR-1 are functional and localized to postsynaptic elements when expressed in nematodes [50]. GLR-1 is required in the interneuron circuit that regulates backward movement and direction reversal, a key element in the foraging behavior of animals. Mutants with depressed GLR-1 synaptic levels rarely reverse direction, whereas mutants with elevated synaptic levels reverse direction with increased frequency [47]–[49], [51], [52]. The synaptic abundance of GLR-1 is regulated by a combination of clathrin-dependent endocytosis [21], [53], [54] and clathrin-independent endocytosis [55]. Ubiquitination plays several critical roles in regulating GLR-1 trafficking, including the direct ubiquitination of GLR-1 itself on four critical lysines, as well as the ubiquitination of several of the key kinases and signaling molecules that regulate GLR-1 trafficking [21], [53], [56], [57]. Whereas K63-linked ubiquitination specifically has not been implicated in GLR-1 regulation, the *C. elegans* genome does encode several UEV proteins [58], including UEV-1, which can interact *in vitro* with UBC-13 [59] and has been implicated in regulating polyglutamine aggregates in *C. elegans* [60].

Here we identify UEV-1 as a regulator of AMPAR trafficking. We show that *uev-1* mutants have defects in GLR-1 localization and GLR-1-mediated behavior that are consistent with an intracellular endosomal accumulation of GLR-1. UEV-1 is expressed broadly in most tissues, yet regulates GLR-1 trafficking cell autonomously in the command interneurons. The localization of other synaptic proteins to the central synapses of these neurons appears unaffected in *uev-1* mutants. We provide evidence that GLR-1 receptors accumulate in endosomes in *uev-1* mutants via clathrin-independent endocytosis, but likely cannot escape such endosomes to either recycle back to the synaptic membrane or be transported for degradation to MVBs and lysosomes. We show that UEV-1 can bind to *C. elegans* UBC-13, and that *ubc-13* mutants have a similar phenotype to *uev-1* mutants. Our results suggest that UEV-1 and UBC-13 function together in *C. elegans* to regulate GLR-1 trafficking, but that UEV-1 is unlikely to be the sole mediator of global K63-linked polyubiquitination in *C. elegans*.

## Results

### GLR-1 accumulates in *uev-1* mutants

**Figure 1:**
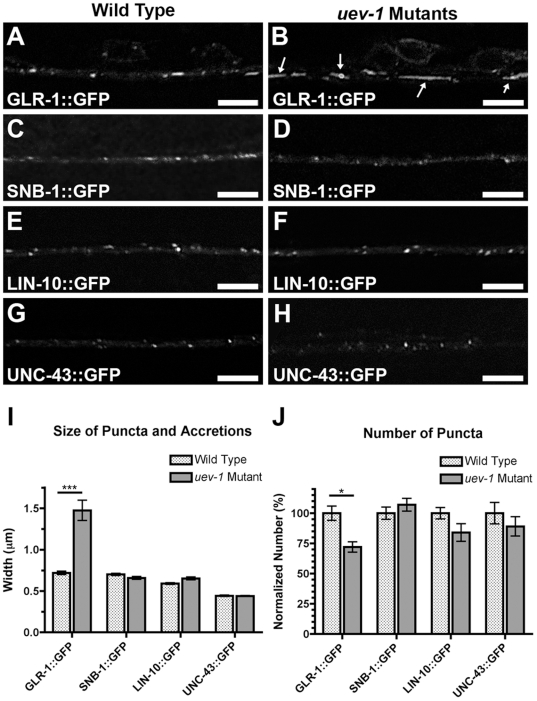
UEV-1 regulates GLR-1 Trafficking. The fluorescence of (A, B) GLR-1::GFP (GluR), (C, D) SNB-1::GFP (synaptobrevin), (E, F) LIN-10::GFP (Mint2), and (G, H) UNC-43::GFP (CaMKII) was observed along ventral cord neurites of (A, C, E, G) wild-type or (B, D, F, H) *uev-1(od10)-*mutant animals. The mean size (I) of fluorescent puncta and accretions (arrows) combined are plotted for adult nematodes of the given genotype (stippled bars for wild type, gray bars for *uev-1* mutants) and for the given fluorescent reporter (indicated below the graph). (J) The mean density (number per 10 microns of ventral cord length) of fluorescent puncta is plotted for adults of the given genotype. For each genotype, the values have been normalized to the mean value of the wild-type control. Whereas wild-type animals have small GLR-1::GFP puncta, *uev-1* mutants accumulate GLR-1::GFP in large accretions (arrows); other synaptic proteins appear not to be affected in *uev-1* mutants. Bar, 5 microns. Error bars are SEM. N = 20–30 animals for each genotype. *P<0.01, ***P<0.001 by ANOVA with Bonferroni multiple comparison tests (only shown for wild type versus mutant for each reporter).

**Figure 2:**
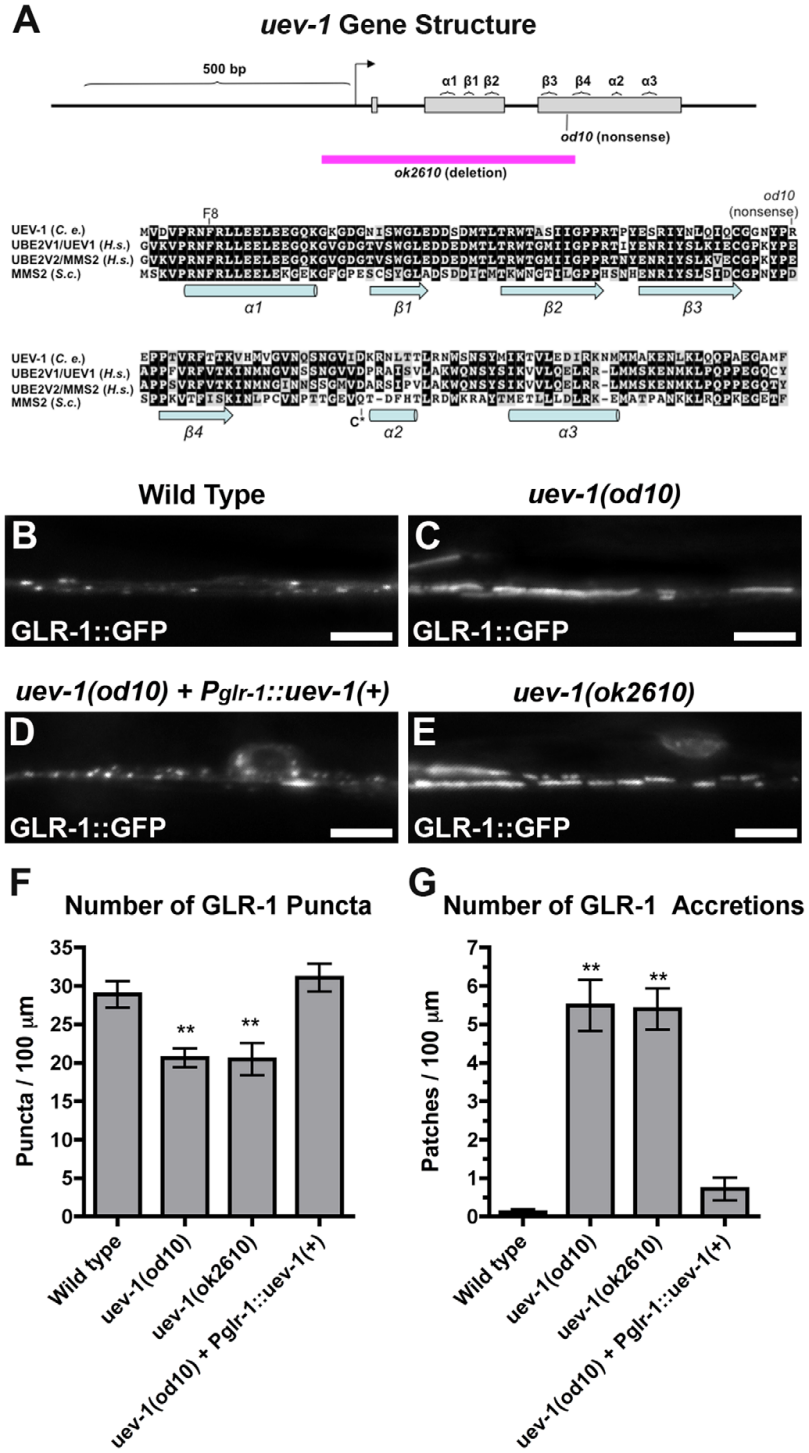
UEV-1 is a member of the E2 ubiquitin-conjugating enzyme variant family. (A) The intron/exon structure of *uev-1* based on sequenced cDNAs is shown in the top panel. Gray boxes indicate exonic coding sequences. The arrow indicates the start of transcription. The site of the *od10* molecular lesion is indicated. Predicted alpha helices and beta sheets, based on alignment with human MMS2, are indicated [99]. The purple line indicates the sequences that are removed by the *ok2610* deletion. The bottom panel shows the amino acid alignment of *C. elegans* (C.e.) *uev-1* with its two putative homologs in humans (H.s.), UBE2V1/UEV1 and UBE2V2/MMS2, along with Mms2 from yeast (S.c.). Black highlighting indicates identity, and gray indicates similarity. The locations of the F8 residue (mutated in our binding constructs) and the residue altered in *od10* to nonsense are indicated. Predicted alpha helices and beta sheets are indicated below the aligned sequence. (B–E) GLR-1::GFP fluorescence in the ventral cord from (B) wild type, (C) *uev-1(od10)* homozygotes, (D) *uev-1(od10)* containing the *P_glr-1_::uev-1(+)* transgene, and (E) *uev-1(ok2610)* homozygotes. The mean number of (F) GLR-1::GFP puncta and (G) GLR-1::GFP accretions per 100 micron of ventral cord length is indicated for the given genotypes. Cell autonomous expression of wild-type *uev-1* via the *glr-1* promoter is sufficient to rescue *uev-1* mutants. Bar, 5 microns. Error bars are SEM. N = 20–30 animals for each genotype. **P<0.01 by ANOVA with Dunnett's multiple comparison to wild type.

We performed a genetic screen for mutants with defects in the localization of GLR-1 receptors tagged with green fluorescent protein (GLR-1::GFP). GLR-1::GFP receptors are normally localized to postsynaptic clusters at neuron–neuron synapses within *C*. *elegans* ventral cord neurites [21], [50], [61]. In wild-type animals, these receptor clusters are generally seen as small (∼0.5–0.7 micron) puncta (Figure 1A). We identified an allele, *od10*, of a gene that we determined to be *uev-1* (please see *Materials and Methods* for details on mapping and cloning). Mutants for *uev-1(od10*) accumulate large, elongated accretions (∼4–6 microns) of GLR-1::GFP, especially in the retrovesicular ganglion region of the ventral nerve cord immediately posterior to the nerve ring (Figure 1B). The average size of puncta and accretions taken together in *uev-1* mutants is twice that of wild-type animals (Figure 1I), and there is a slight decrease in the number of GLR-1 puncta (normalized for neurite length; Figure 1J). We also analyzed GLR-1 accretions alone by identifying them based on their size and elongated morphology. Whereas wild-type animals nearly lack these accretions, *uev-1* mutants have significant numbers of them (Figure 2G). These results indicate that UEV-1 is involved in the regulation of GLR-1-containing glutamate receptors in ventral cord neurites.

The change in the pattern of GLR-1 localization could be a consequence of *uev-1* mutants having general defects in protein trafficking or in synapse formation. To test this possibility, we examined the localization of other synaptic proteins: SNB-1 (synaptobrevin), UNC-43 (CaMKII), and LIN-10 (Mint2). We used transgenes that express GFP-tagged versions of these proteins using the *glr-1* promoter [50], [61]-[63]. We introduced these transgenes into wild type and *uev-1* mutants to observe in their ventral cord neurites the subcellular localization of the proteins they encode. SNB-1::GFP is localized to presynaptic terminals [50], [64], and we found no significant change in the size or number of SNB-1::GFP-labeled terminals in *uev-1* mutants compared with wild type (Figure 1, C, D, I, and J). UNC-43::GFP and LIN-10::GFP colocalize with GLR-1 at postsynaptic elements in the ventral cord [61]-[63], and we found no significant difference in the size or number of either LIN-10::GFP (Figure 1, E, F, I, and J) or UNC-43::GFP (Figure 1, G–J) puncta in *uev-1* mutants compared to wild type. These results lead us to conclude that the aberrant accumulation of GLR-1 in *uev-1* mutants is not because of gross defects in synaptic protein trafficking or in the formation of synapses to GLR-1-expressing neurons. In addition, because the *glr-1* promoter was used to express all of these synaptic proteins, it is unlikely that the defects observed in *uev-1* mutants are due to an elevation in *glr-1* transcription.

### UEV-1 encodes a member of the UEV family of proteins

The *uev-1* gene contains three exons that encode a 139 amino acid protein in the UEV family of proteins. UEV proteins are well conserved across species as diverse as yeast (*S. cerevisiae)*, nematodes (*C. elegans)*, and humans (Figure 2A) [31]–[34]. We identified *od10* as an allele of *uev-1* by genetic mapping and transformation rescue using the F39B2 cosmid, which contains *uev-1* under its own promoter (data not shown). We confirmed this, as well as the fact that UEV-1 acts cell autonomously, by transformation with a construct containing *uev-1* coding sequences under the *glr-1* promoter (Figure 2B–G). Following our identification of *od10* as an allele of *uev-1*, a new deletion allele, *ok2610*, became available from the *C. elegans* Genome Consortium. To determine the molecular nature of these alleles, we sequenced genomic DNA from both *uev-1* mutants. The *od10* mutation alters the UEV-1 protein sequence from an arginine to an opal stop codon at amino acid 70, while the *ok2610* deletion removes an approximately 500 base pair region that includes the first two exons of *uev-1* and the beginning of the third (Figure 2A and Figure S1).

We hypothesized that the phenotype observed in *od10* homozygotes was likely due to a complete or severe loss of UEV-1 function, as half of the C-terminus of the protein is predicted to be absent. To confirm that the *uev-1(od10)* phenotype is due to loss of UEV-1 function, we also examined GLR-1 localization in *uev-1(ok2610)* and found that it was virtually identical in appearance, number of puncta, and number of accretions observed in *uev-1(od10)* mutants (Figure 2C, E, F, and G). Since *uev-1(ok2610)* mutants are deleted for DNA encoding the N-terminus of UEV-1, as well as the promoter and start of transcription, we conclude that both *od10* and *ok2610* are likely to be null alleles of *uev-1*.

### UEV-1 is broadly expressed and affects motoneuron development

**Figure 3:**
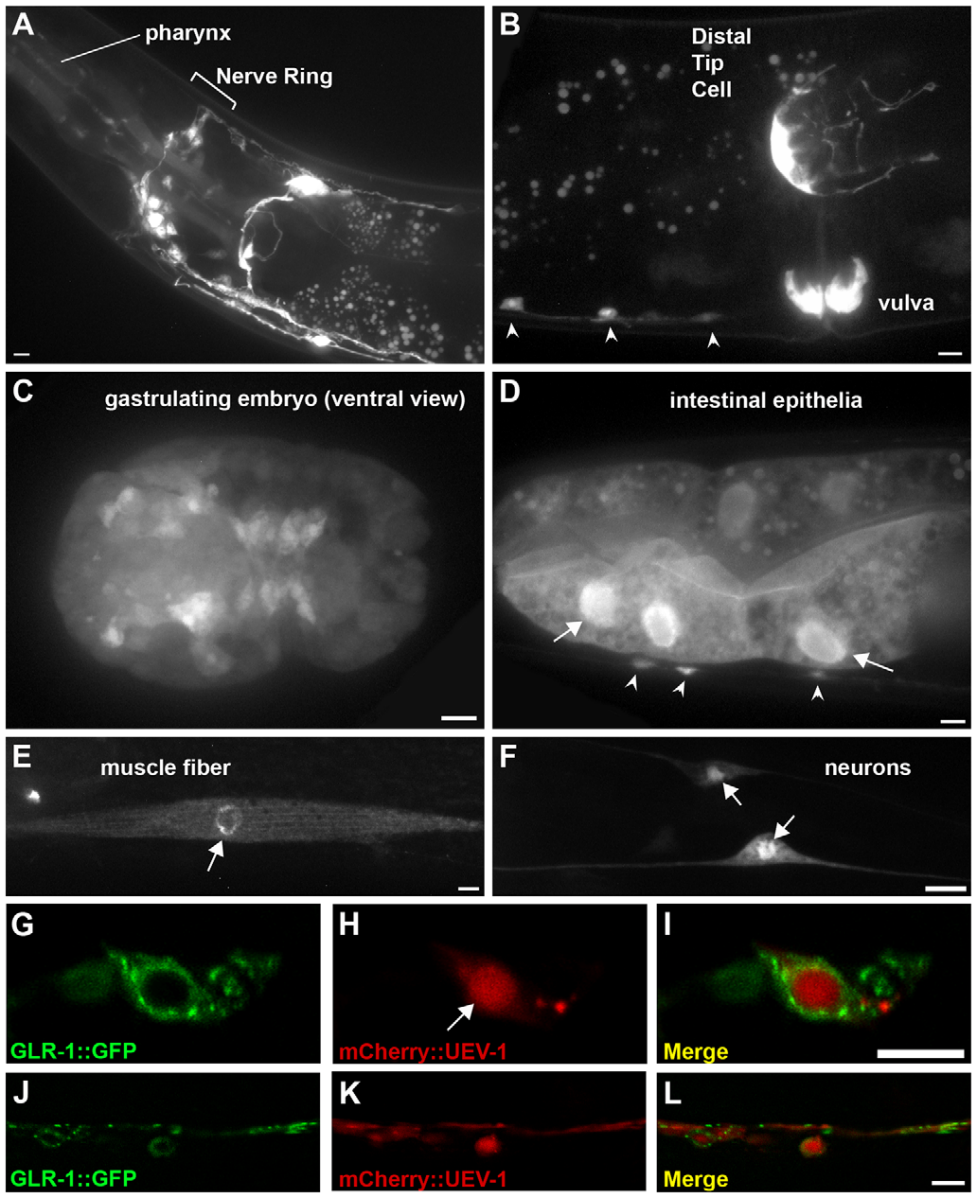
UEV-1 is broadly expressed. (A–F) Fluorescence from animals transgenic for *P_uev-1_::uev-1::gfp*. Expression is detected in (A) pharynx and multiple head neurons, (B) distal tip cell, vulval epithelia, and ventral cord motoneurons (arrowheads), (C) most cells of embryos (gastrulating embryo shown from ventral view), (D) intestinal epithelia and motoneurons (arrowheads), (E) body wall muscle, and (F) ventral cord motoneurons. (D–F) Arrows indicate nuclear enrichment of the UEV-1::GFP protein. (G–L) Fluorescence from *uev-1(od10)*-mutant animals co-expressing (G, J) GLR-1::GFP and (H, K) mCherry::UEV-1 via the *glr-1* promoter. (I, L) Merged images. (G–I) UEV-1 is enriched in nuclei (arrow), but can be found at punctate structures in the cell body cytoplasm (PVC neuron cell body is shown). (J–L) UEV-1 is uniformly distributed along ventral cord neurites. Bar, 5 microns.

In addition to the GLR-1 mislocalization phenotype, we observed that *uev-1* mutants have a locomotion defect, flexing their bodies more than wild-type nematodes as they move, suggesting that UEV-1 might function in multiple neuron types. To determine where UEV-1 might function, we created a transgenic reporter containing 2 kilobases of upstream *uev-1* promoter sequences and the entire *uev-1* transcription unit fused in frame to GFP. We introduced the resulting *P_uev-1_::uev-1::gfp* reporter into the *C. elegans* germline, and observed that UEV-1 is broadly expressed in the pharynx, neurons, muscle cells, vulva, embryos, intestine, and in the anus/tail (Figure 3A–F). We found strong expression in the distal tip cell (Figure 3B), which is important in development for gonad morphology [65], but upon examination we found that the gonads of *uev-1* mutant worms were normal in shape (data not shown). We also found expression in the motoneurons that line the ventral cord (Figure 3B, D, F). We noted that the UEV-1::GFP chimeric protein is localized diffusely throughout the cytosol of most cells, but with some enrichment in the nucleus (Figure 3D–F).

To examine UEV-1 subcellular localization specifically in the command interneurons, we generated a transgene containing the *glr-1* promoter sequences driving sequences encoding the fluorescent protein mCherry fused to the complete UEV-1 reading frame sequences. We introduced the *P_glr-1_::mCherry::uev-1* transgene into nematodes expressing GLR-1::GFP, and observed that mCherry::UEV-1 protein is enriched in neuronal nuclei (Figure 3G-I). As observed in the cytosol for UEV-1::GFP, we find that mCherry::UEV-1 is diffusely distributed throughout ventral cord neurites (Figure 3J–L).

**Figure 4:**
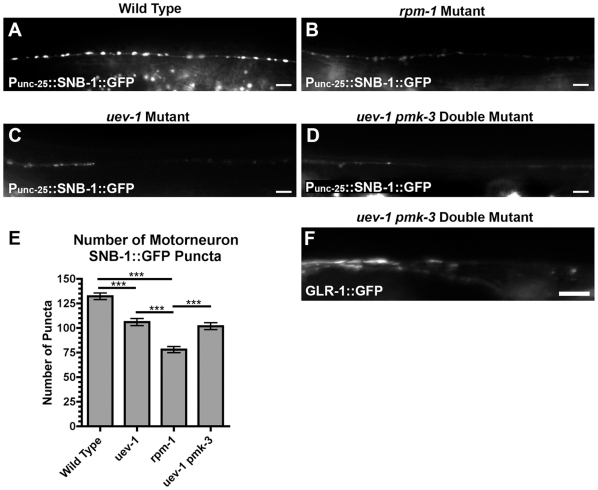
UEV-1 is required for motoneuron synaptic bouton differentiation. (A–D) Fluorescence from animals transgenic for *P_unc-25_::snb-1::gfp* (synaptobrevin). (A) Presynaptic boutons labeled with SNB-1::GFP are distinct along the dorsal and ventral cords of wild-type animals. Such boutons are missing, irregular in shape and size, and/or abnormally spaced in (C) *uev-1(od10)* mutants, a phenotype reminiscent of that observed in (B) *rpm-1* mutants. (D) Mutations in *pmk-3* do not alter the *uev-1* phenotype. (E) The total number of dorsal cord SNB-1::GFP puncta is plotted for the indicated genotypes. (F) GLR-1::GFP accumulation in accretions in *uev-1 pmk-3* double mutants is similar to that observed in *uev-1(od10)* single mutants. Bar, 5 microns. Error bars are SEM. N = 20–30 animals for each genotype. ***P<0.001 by ANOVA with Bonferroni multiple comparison tests.

Given the locomotion defect and the motoneuron expression, we hypothesized that UEV-1 might play a role in regulating motoneuron synapse formation. We examined the subcellular localization of SNB-1 (synaptobrevin) using a transgene, *juIs1[P_unc-25_::SNB-1::GFP]*, that expresses a SNB-1::GFP chimeric protein in the GABAergic motor neurons [66]. In wild-type animals, SNB-1::GFP is localized to large neuromuscular junction (NMJ) boutons regularly spaced along both the dorsal and ventral cords (Figure 4A) [66]. In *uev-1(od10)* mutants, we observed a decreased number of SNB-1::GFP NMJ boutons and an irregularity in interbouton spacing, similar to the phenotype observed in *rpm-1* mutants (Figure 4B, C) [67]. We quantified the number of dorsal cord SNB-1::GFP boutons in *uev-1(od10)* mutants, and found it to be significantly less than that seen in wild-type animals, although not as severely depressed as in *rpm-1* nulls (Figure 4E).

RPM-1 is an E3 ubiquitin ligase that directly ubiquitinates the DLK-1 kinase, which is part of the PMK-3/p38 MAPK signaling pathway. RPM-1 is thought to regulate presynaptic bouton formation by repressing PMK-3 activity [68]. RPM-1 also acts in the command interneurons to regulate GLR-1 endocytosis by repressing PMK-3 activity [56]. Indeed, both the motoneuron NMJ phenotype and the interneuron GLR-1-trafficking phenotype observed in *rpm-1* mutants are suppressed by loss of function mutations in *pmk-3* [56], [68]. We tested if either the NMJ bouton defect or the GLR-1 accumulation defect is suppressed in animals mutated for both *pmk-3* and *uev-1*, but detected no suppression of either defect (Figure 4D–F). We conclude that UEV-1 can regulate either presynaptic or postsynaptic differentiation depending on the specific neuron type, similar to what has been observed for RPM-1. However, the regulatory targets of UEV-1 are likely to be distinct from those of RPM-1.

### UEV-1 regulates GLR-1 trafficking independent of the ERAD pathway

**Figure 5:**
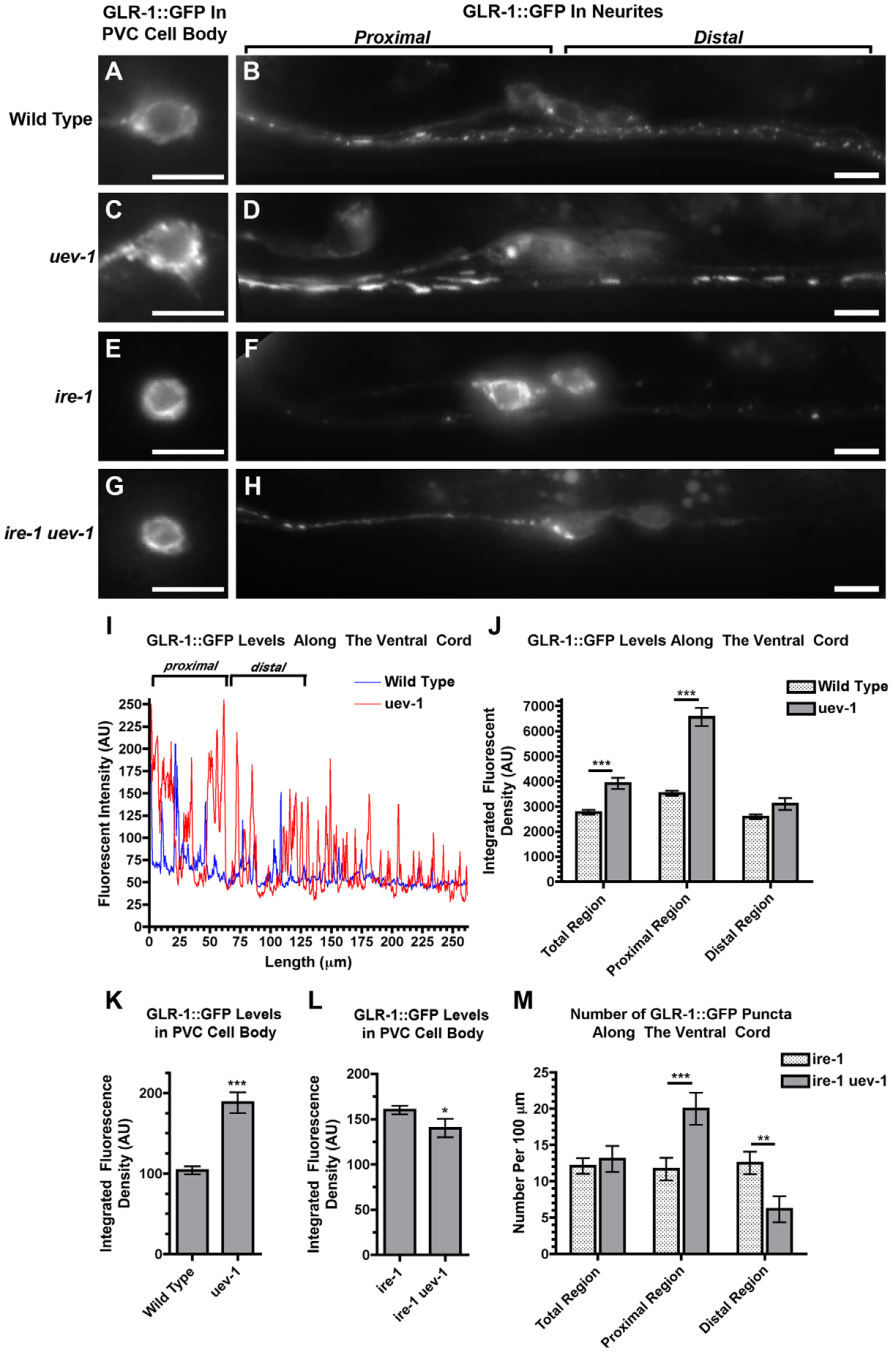
UEV-1 regulates both GLR-1 levels and spatial distribution. (A–H) GLR-1::GFP fluorescence in (A, C, E, G) neuron cell bodies and (B, D, F, H) along the ventral cord for (A, B) wild type, (C, D) *uev-1(od10)* mutants, (E, F) *ire-1* mutants, and (G, H) *ire-1 uev-1* double mutants. To quantify the unusual spatial distribution of GLR-1 in *uev-1* mutants, the region 65 microns anterior (left in the figure) of the RIG/AVG cell bodies (these neurons contribute little GLR-1::GFP to the ventral cord, used here solely as a landmark) was considered as “proximal” to the neuron cell bodies of the command neurons in the head (AVA, AVB, AVD, AVE, and PVC, which contribute almost all of the GLR-1::GFP along the ventral and are outside of the images in this figure), whereas the region 65 microns posterior (right in the figure) of the RIG/AVG cell bodies were considered as “distal” to the command neuron cell bodies. The images in E–H were collected at several times the exposure as the images in A–D as no single exposure time possessed the dynamic range to precisely capture all four genotypes. (I) Line profiles for GLR-1::GFP fluorescent intensity along the ventral cord for a wild-type animal (blue) and a *uev-1(od10)* mutant (red) after removal of background autofluorescence. Baseline is a measure of unlocalized GLR-1::GFP levels. Brighter GLR-1::GFP puncta are observed more proximal along the neurites to the cell bodies. This proximal-distal bias is increased in *uev-1* mutants compared to wild type. (J) Mean GLR-1::GFP fluorescence (sum of pixel values) along the ventral cord for either the total cord, the proximal region, or the distal region for wild type (stippled) or *uev-1* mutants (gray). (K, L) GLR-1::GFP fluorescence (sum of pixel values) observed in the PVC neuron cell bodies for the indicated genotypes. (M) Mean number of GLR-1::GFP puncta along the ventral cord (per 100 micron length) for either the total cord, the proximal region, or the distal region for wild type (stippled) or *uev-1(od10)* mutants (gray). Bar, 5 microns. Error bars are SEM. N = 20–30 animals for each genotype. (J, M) **P<0.01, ***P<0.001 by ANOVA with Bonferroni multiple comparison tests (only shown for wild type versus mutant for each reporter). (K, L) *P<0.01, ***P<0.0001 by t-test.

One possible explanation for the accumulation of GLR-1 in *uev-1* mutants could be that UEV-1 contributes to the Endoplasmic Reticulum Associated Protein Degradation (ERAD) pathway. This pathway targets misfolded proteins in the ER for ubiquitination, export into the cytoplasm, and subsequent degradation by the 26S proteasome [69]–[71]. Consistent with this possibility, we also observed that the cell bodies of *uev-1* mutants have higher levels of GLR-1::GFP than those observed in wild-type animals (Figure 5A, C, K).

A closely related pathway, the Unfolded Protein Response (UPR), regulates the expression of ER resident chaperones that assist in dealing with ER stress [72], [73]. GLR-1 requires proper UPR signaling, even in the absence of stress, to exit the ER [74]. In mutants for the UPR gene *ire-1*, GLR-1::GFP becomes trapped in the ER, visible as a “donut” of fluorescence (Figure 5E) surrounding neuronal nuclei, while very little GLR-1 is found in puncta along the ventral nerve cord (Figure 5F) [74]. In addition, the level of GLR-1 in the cell bodies of *ire-1* mutants is lower than that in wild-type cell bodies, suggesting that much of the ER-trapped receptor in *ire-1* mutants is removed by ERAD [74], [75]. We reasoned that if UEV-1 regulates GLR-1 as part of ERAD, then in the absence of both UEV-1 and UPR function, GLR-1 receptors that would normally have been degraded would instead accumulate to higher levels in the ER. To address this question, we captured fluorescent images of cell bodies from wild type, *uev-1* single mutants, *ire-1* single mutants, and *uev-1 ire-1* double mutants. While we could not find a single microscope setting with the dynamic range to quantify all four genotypes relative to each other, we were nevertheless able to compare *uev-1* single mutants to wild type, and *uev-1 ire-1* double mutants to *ire-1* single mutants. We found that, unlike *uev-1* single mutants, which have elevated cell body levels of GLR-1 relative to wild type (Figure 5C, K), instead *uev-1 ire-1* double mutants have slightly decreased levels of GLR-1 relative to *ire-1* single mutants (Figure 5G, L). Our results suggest that mutations like *ire-1* that trap GLR-1 receptors in the ER preclude those receptors from regulation by UEV-1, suggesting that UEV-1 regulates GLR-1 trafficking at a step after the ER, making it unlikely that UEV-1 is part of the ERAD pathway.

An alternative possibility for how UEV-1 regulates GLR-1 is that it regulates trafficking at a step further down in the secretion pathway, perhaps by mediating GLR-1 turnover after endocytosis. Indeed, when we analyzed the fluorescence levels of GLR-1::GFP in profile along the ventral cord, we found that the unlocalized baseline in wild type and *uev-1* mutants is similar, but that the peaks of GLR-1::GFP fluorescence are higher and more frequent in *uev-1* mutants compared to wild-type animals (Figure 5B, D, I). We also noticed a spatial asymmetry in the *uev-1* mutant phenotype, with GLR-1 accretions occurring more anterior along the ventral cord and hence more proximal to the neuron cell bodies (Figure 5I, J; Figure S2 for overview of ventral cord spatial organization). As described above, mutations in *ire-1* dramatically reduce the amount of GLR-1 that reaches ventral cord neurites (Figure 5F). Interestingly, while double mutants for both *ire-1* and *uev-1* have similar levels of GLR-1 in their cell bodies compared to *ire-1* single mutants, they have elevated levels of GLR-1 in their ventral cord neurites along the same proximal region at which GLR-1 accretions accumulate in *uev-1* single mutants, and decreased GLR-1 at more distal sites (Figure 5H, M). We also examined the levels of *glr-1* mRNA and GLR-1::GFP protein in wild type and *uev-1* mutants, and found no significant difference in their levels between the two genotypes (Figure S3). Taken together, our results indicate that UEV-1 regulates the spatial location at which GLR-1 receptors accumulate along the proximal-distal axis of the neurites rather than just affecting overall GLR-1 protein levels throughout the neuron.

### GLR-1 accumulates at RAB-10-containing endosomes in *uev-1* mutants

**Figure 6:**
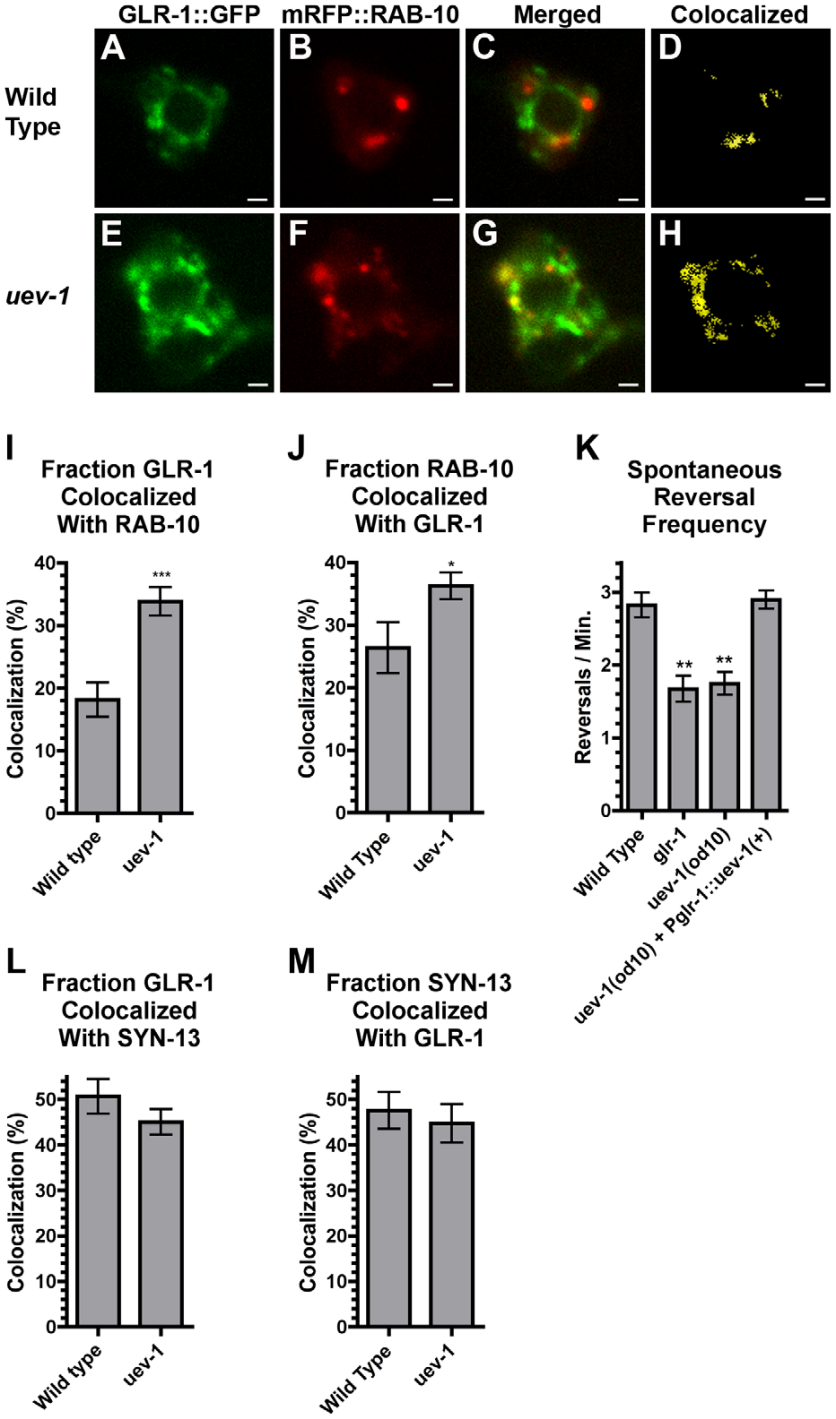
UEV-1 regulates GLR-1 colocalization with endosomal protein RAB-10. (A, E) GLR-1::GFP and (B, F) mRFP::RAB-10 fluorescence was observed in single-plane confocal images of PVC neuron cell bodies from (A–D) wild-type animals and (E–H) *uev-1(od10)* mutants. (C, G) Merged images. (D, H) Binary masks (yellow) were created to highlight pixels with matching intensity values for both GLR-1::GFP and mRFP::RAB-10, indicating colocalization. The mean percent of (I) GLR-1 colocalized with RAB-10, and (J) RAB-10 colocalized with GLR-1 is plotted for the indicated genotypes. More GLR-1 is found colocalized with RAB-10 in *uev-1(od10)* mutants. (K) The mean spontaneous reversal frequency as an indication of GLR-1 function is plotted for the indicated genotypes. The mean percent of (L) GLR-1::GFP colocalized with Syntaxin-13::mRFP, and (M) Syntaxin-13::mRFP colocalized with GLR-1::GFP is plotted for the indicated genotypes. Bar, 1 microns. Error bars are SEM. N = 20–30 animals for each genotype. (I, J) ***P<0.0001, *P<0.05 by t-test. (K) **P<0.01, *P<0.05 by ANOVA with Dunnett's multiple comparison test to wild type.

The GLR-1::GFP accretions that we observed in *uev-1* mutants could represent receptor trapped at an intracellular location, perhaps endosomal in nature [55], [56]. To test this possibility, we examined several endosomal markers, each labeled with mRFP and expressed under the *glr-1* promoter. We coexpressed these with GLR-1::GFP in our wild-type and *uev-1* animals, allowing us to check for colocalization. While these endosomal markers were too dim to observe colocalization in the neurites, we were able to examine colocalization in the neuron cell bodies [55], [76]. We observed an increase in the amount of GLR-1::GFP that colocalized with mRFP::RAB-10 in neuron cell bodies from *uev-1* mutants compared to wild-type animals (Figure 6A–J); RAB-10 is a small Rab GTPase that associates with the early endosomes that are involved in recycling clathrin-independent cargo [55], [77]. By contrast, we observed no significant difference in the amount of colocalization between GLR-1::GFP and SYN-13::mRFP (Figure 6L, M; Figure S4) or RME-1::mRFP (data not shown), which are associated with early and recycling endosomes, respectively, and are involved in recycling clathrin-dependent cargo [55], [76], [78]. Given that we see no increase in the levels of GLR-1 colocalization with SYN-13 or RME-1, it is unlikely that the increased colocalization of GLR-1 with RAB-10 observed in *uev-1* mutants is simply due to elevated levels of GLR-1 in the cell body. We confirmed this by independently analyzing colocalization using randomization through a confined displacement algorithm (Figure S4) [79].

If the GLR-1::GFP accretions observed in *uev-1* mutants indicate that receptors are becoming trapped at endosomes, then we would expect a decrease in GLR-1 function in *uev-1* mutants [55], [56]. Normally, GLR-1 functions to regulate changes in locomotion direction. As *C*. *elegans* move, they generally go forward, but will occasionally spontaneously reverse direction; GLR-1 signaling positively induces these spontaneous reversals in locomotion [49], [51]. Mutants with reduced GLR-1 signaling or reduced levels of GLR-1 at the synapse have a lower frequency of spontaneous reversals, whereas mutants with increased GLR-1 signaling or higher levels of cell surface GLR-1 have a higher frequency of spontaneous reversal [21], [47], [48], [51]–[53]. We measured the spontaneous reversal rate of *uev-1* mutants, and found that the rate was significantly lower in *uev-1* mutants than in wild type, similar to *glr-1* mutants, which entirely lack GLR-1 (Figure 6K). Expression of a wild-type *uev-1* minigene via the *glr-1* promoter is sufficient to rescue this behavioral defect. Therefore, little receptor appears to be functioning at the postsynaptic membrane in *uev-1* mutants. Instead, it is likely that a large amount of the receptor is in an intracellular compartment, possibly endosomal in nature.

### UEV-1 regulates GLR-1 trafficking independent of clathrin-mediated endocytosis

**Figure 7:**
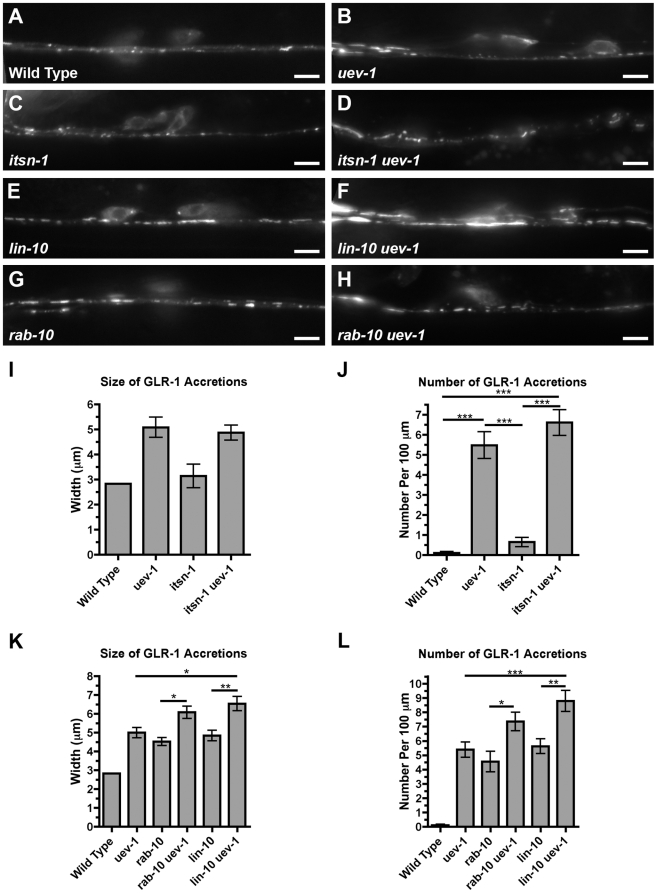
UEV-1-dependent trafficking of GLR-1 is independent of clathrin-mediated endocytosis. (A-H) GLR-1::GFP ventral cord fluorescence is shown for the indicated genotypes: (A) wild type, (B) *uev-1*, (C) *itsn-1*, (D) *itsn-1 uev-1,* (E) *lin-10*, (F) *lin-10 uev-1*, (G) *rab-10*, and (H) *rab-10 uev-1*. The *uev-1(ok2610)* allele was used in these experiments. Mean (I, K) size and (J, L) number of GLR-1::GFP accretions observed along the ventral cord of the indicated genotypes. Bar, 5 microns. Error bars are SEM. N = 20–30 animals for each genotype. ***P<0.001, **P<0.01, *P<0.05 by ANOVA with Bonferroni multiple comparison tests. For (K) and (L), only comparisons between double mutants and their corresponding single mutants are shown for clarity.

GLR-1 is endocytosed by a combination of clathrin-dependent and clathrin-independent mechanisms, with the PDZ protein LIN-10 mediating the recycling of receptors endocytosed by the former mechanism, and RAB-10 mediating the recycling of receptors endocytosed by the latter mechanism (Figure S6) [55], [77]. In *uev-1* mutants, GLR-1 is colocalized with RAB-10, suggesting that it might regulate GLR-1 recycling along the clathrin-independent pathway, and that the accumulation of GLR-1 that occurs in *uev-1* mutants does not occur through clathrin-mediated endocytosis. To examine this possibility, we used the *itsn-1* mutation, which impairs the function of the ITSN-1 Intersectin protein – an adaptor that helps mediate the clathrin-dependent endocytosis of GLR-1 [55], [80]–[84]. We introduced an *itsn-1* mutation into *uev-1* mutants to determine whether it would suppress the accumulation of GLR-1 in accretions. We found that *itsn-1* did not have any significant effect on either the average size or number of accretions in *uev-1* animals (Figure 7A–D, I–J). We had previously generated a transgene, *P_glr-1_::rfp::rab-5(GDP),* which contains a mutated form of the *rab-5* cDNA that can suppress the clathrin-dependent endocytosis of GLR-1 [56]. We expressed this transgene in both wild-type and *uev-1* animals, but as with the *itsn-1* experiment, this did not result in the suppression of the *uev-1* mutant phenotype (data not shown), suggesting that UEV-1 regulates GLR-1 trafficking at a step independent of clathrin-mediated endocytosis.

As LIN-10 and RAB-10 mediate the recycling of GLR-1 receptors along the clathrin-dependent and clathrin-independent endocytosis pathways, respectively, double mutants for *lin-10* and *rab-10* show an additive effect on GLR-1 accumulation by simultaneously blocking both pathways [55]. Given that UEV-1 does not appear to be downstream of clathrin-dependent endocytosis, and that GLR-1 colocalizes with RAB-10 in *uev-1* mutants, we reasoned that UEV-1 might function in the same pathway as RAB-10. To test this possibility, we made double mutant strains of *uev-1* with either *lin-10* or *rab-10* null mutations. We found that in a *lin-10 uev-1* double mutant, there was a significant increase in GLR-1 accretion size and number compared to either single mutant alone (Figure 7E, F, K, L), making it likely that LIN-10 and UEV-1 do not work in the same pathway. However, we found that in a *rab-10 uev-1* double mutant, there was a significant increase in GLR-1 accretion size and number only compared to the *rab-10* single mutant, but not compared to the *uev-1* single mutant alone (Figure 7G, H, K, L). This suggests that UEV-1 and RAB-10 might function in the same pathway, with the more severe phenotype in *uev-1* mutants indicating the possibility that UEV-1 also works in an additional pathway.

### UEV-1 and ubiquitin-mediated turnover of GLR-1

**Figure 8:**
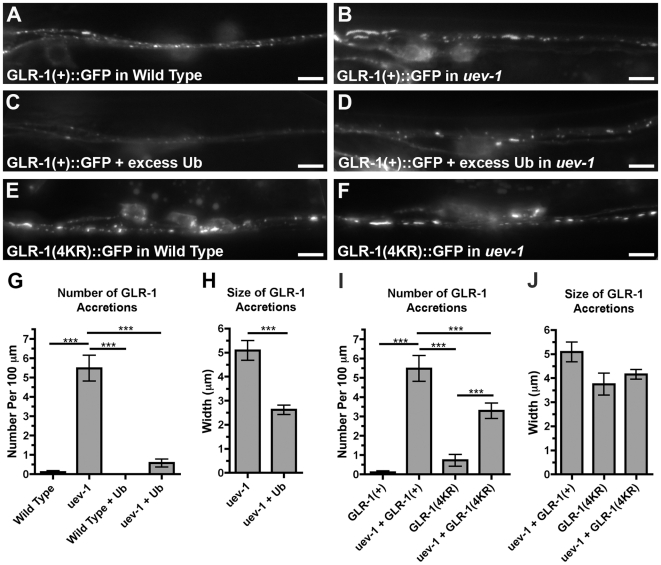
UEV-1 and ubiquitin-mediated turnover of GLR-1. (A–D) GLR-1::GFP ventral cord fluorescence in (A) wild type, (B) *uev-1(od10)* mutants, (C) wild type that overexpress free ubiquitin, and (D) *uev-1(od10)* mutants that overexpress free ubiquitin. (E, F) GLR-1(4KR)::GFP ventral cord fluorescence in (E) wild type and (F) *uev-1(od10)* mutants. The mean (G, I) number and (H, J) size of GLR-1::GFP accretions is plotted for the indicated genotypes. In (I–J), “GLR-1(+)” indicates the presence of the wild-type GLR-1::GFP-expressing transgene, whereas “GLR-1(4KR)” indicates the presence of the GLR-1(4KR)::GFP-expressing transgene; GLR-1(4KR)::GFP cannot be ubiquitinated. Bar, 5 microns. Error bars are SEM. N = 20–30 animals for each genotype. ***P<0.001 by ANOVA with Bonferroni multiple comparison tests.

The ubiquitination of GLR-1, like many membrane receptors, is necessary for its endocytosis [19]–[22]. GLR-1 ubiquitination and hence turnover can be accelerated by the overexpression of ubiquitin monomers from a transgene containing the *glr-1* promoter, resulting in reduced number and size of GLR-1::GFP puncta (Figure 8A, C) [21]. We introduced this *P_glr-1_::ubiquitin* transgene into *uev-1* mutants and found that overexpression of ubiquitin can decrease the number and size of GLR-1 accretions in *uev-1* mutants (Figure 8D, G, H). However, *uev-1* mutations partially block the turnover of GLR-1 caused by increased ubiquitin levels (Figure 8D,G), suggesting that UEV-1 is required for one or more ubiquitin-mediated processes that regulate GLR-1 trafficking.

GLR-1 ubiquitination occurs at four critical lysines on the intracellular tail of the receptor, and expression of GLR-1(4KR)::GFP, a mutant version in which each of the four lysine residues has been changed to arginine to preclude direct receptor ubiquitination, results in an increase in the number and size of GLR-1 puncta along the ventral cord because of decreased endocytosis (Figure 8E) [21]. There is also a slight increase in the number of accretions relative to wild type (Figure 8I). We introduced the *P_glr-1_::glr-1(4kr)::gfp* transgene into *uev-1* mutants to determine whether the phenotype of *uev-1* was directly dependent on receptor ubiquitination. We found that GLR-1(4KR)::GFP, like wild-type GLR-1::GFP, accumulates in accretions in *uev-1* mutants (Figure 8F, I, J), although with some reduction in number. Furthermore, expression of GLR-1(4KR)::GFP in *uev-1* mutants does not suppress the low reversal rate observed in *uev-1* mutants containing wild-type GLR-1::GFP (data not shown). Taken together, these data indicate that (1) preventing the ubiquitination of GLR-1 tails cannot mimic the *uev-1* mutant phenotype, and (2) the ubiquitination of GLR-1 itself does not seem to alter the internalized accumulation of receptors in *uev-1* mutants dramatically. Thus, UEV-1 probably regulates the ubiquitination of other proteins that in turn regulate GLR-1 trafficking.

### UEV-1 might function by regulating the K63-linked ubiquitination state of one or more substrates

UEV-1 is similar to other UEV proteins in a variety of organisms (Figure 2A), including the ESCRT protein Vps23 [33], [85]. ESCRT proteins are important for routing ubiquitinated receptors from the endosome to multivesicular bodies and lysosomes, and Vps23 is a subunit of ESCRT-1, which binds ubiquitinated receptors in the early endosome [36]. We reasoned that if UEV-1 is working as part of the ESCRT complex, then mutants for homologs of other ESCRT complex genes or related vesicular trafficking proteins might have a GLR-1::GFP localization phenotype similar to that seen in *uev-1*. However, when we introduced *P_glr-1_::glr-1::gfp* into several of these mutants, including *alx-1* [86] and *stam-1* [87], [88], we found that GLR-1:GFP localization was similar to that found in wild type (data not shown).

**Figure 9:**
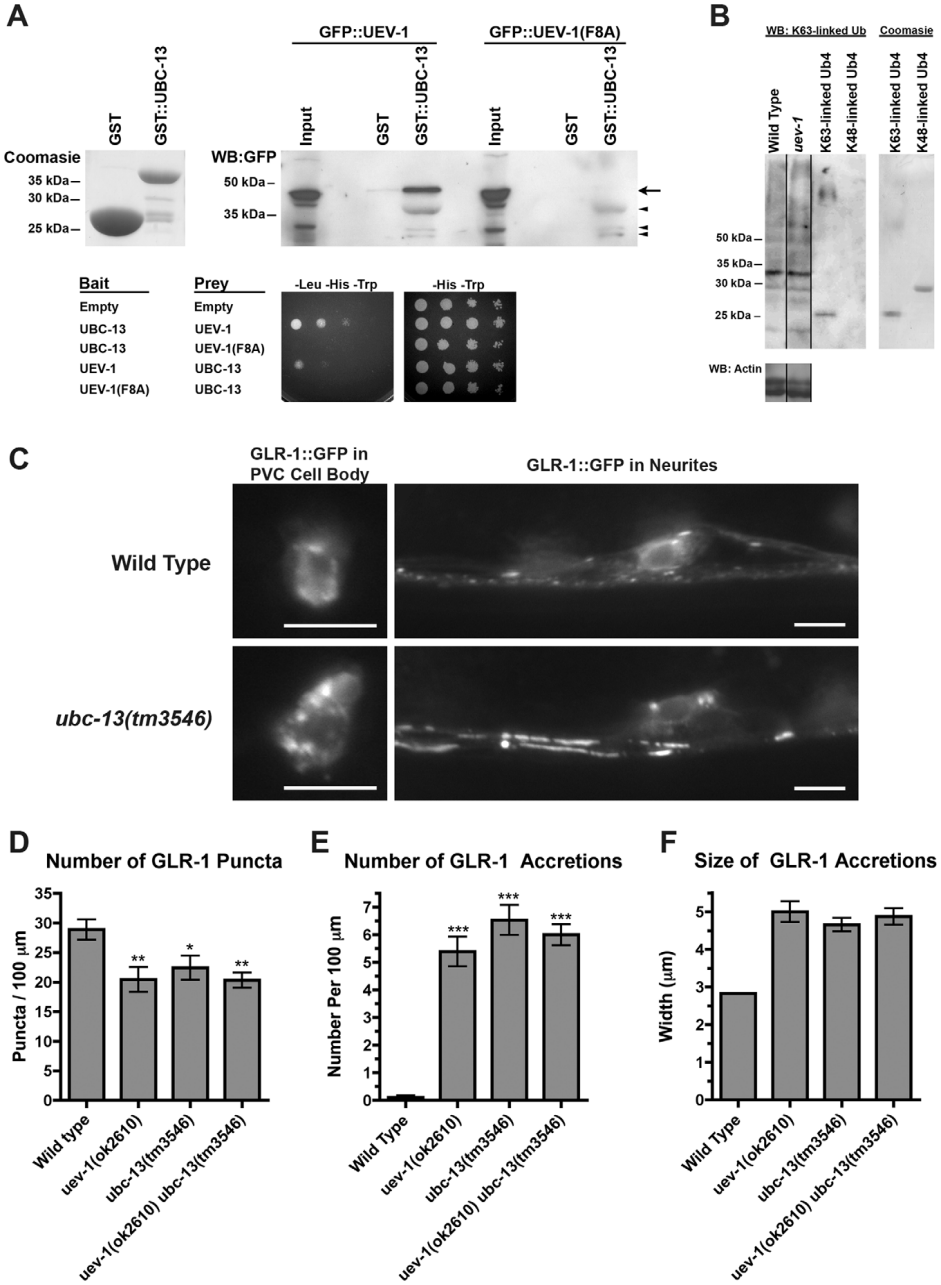
UEV-1 interacts with UBC-13 to regulate GLR-1. (A) Top left panel shows a Coomasie-stained SDS-PAGE for bacterially produced GST and GST::UBC-13 used in the pull down assay. Top right panel shows a Western blot using anti-GFP antibodies to detect GFP::UEV-1 or GFP::UEV-1(F8A) pulled down from COS7 lysates using either GST or GST::UBC-13 bound to beads. “Input” indicates 10% of the lysate used in the binding reaction. Arrow indicates the specific GFP::UEV-1 protein. Arrowheads indicate non-specific bands in the lysate that are pulled down by GST::UBC-13 and detected on the Western blot. Bottom panel shows growth on media either selecting for interaction (–Leu –His –Trp) or allowing growth without selection (–His –Trp) for 10 fold serial dilutions of yeast cultures co-expressing the indicated bait and prey plasmids. Similar results were found in 3 independent experiments. (B) Western blots for K63-linked polyubiquitinated proteins or actin as a loading control. Purified tetra-ubiquitin (Ub4) is present on the SDS-PAGE in either the K63-linked form (which runs at around 25 kDa) or the K48-linked form (which runs at around 30 kDa). Coomasie-stained SDS-PAGE for each tetra-ubiquitin protein is also shown. Similar results were found in 5 independent experiments. (C) GLR-1::GFP fluorescence from either cell bodies (left hand panels) or neurites around the retrovesicular region (right hand panels) are shown for the indicated genotypes. Like *uev-1* mutants, *ubc-13(tm3546)* mutants accumulate GLR-1::GFP in their cell bodies and at proximal regions along their ventral cord neurites. Bar, 5 microns. The (D) number of GLR-1::GFP puncta, (E) number of GLR-1::GFP accretions, and (F) size of GLR-1::GFP accretions are indicated for the given genotypes. *P<0.05, **P<0.01, ***P<0.001 by ANOVA with Dunnett's comparison to wild type. Error bars are SEM. N = 20–30 animals for each genotype.

UEV proteins in other organisms can function by forming heterodimers with Ubc13 so as to catalyze the formation of K63-linked polyubiquitin chains. We performed two different assays to test whether *C. elegans* UEV-1 and UBC-13 can physically interact. First, we expressed either GST or GST::UBC-13 in bacteria, and then tested the ability of these proteins when bound to glutathione agarose beads to pull down GFP-tagged UEV-1 from lysates of transfected COS7 cells. We found that GST::UBC-13 specifically pulls down GFP::UEV-1 (Figure 9A). The binding between Mms2 (a yeast homolog of UEV-1) and Ubc13 can be disrupted by mutating the eighth residue (Phenylalanine) of Mms2 to Alanine [89]. We introduced this change into the corresponding residue of GFP::UEV-1, and found that it abolished the interaction with GST::UBC-13 (Figure 9A). Similar results were observed in five different pull down experiments. Second, we introduced UBC-13 and UEV-1 into bait and prey vectors for yeast two-hybrid, using growth on –Leu plates as an indicator of an interaction. We found that wild-type UEV-1, but not UEV-1 with the F8A mutation, interacted with UBC-13 regardless of which protein was bait and which was prey (Figure 9A). Our results indicate that *C. elegans* UEV-1 and UBC-13 can interact in a similar fashion as their homologs in other species do.

Based on the subtle phenotypes and overall viability and fertility of *uev-1* mutants, we reasoned that UEV-1 might have a smaller, more selective set of targets for K63-linked ubiquitination than would UBC-13, and that other proteins might function with UBC-13 to conduct most K63-linked ubiquitination events in nematodes. To test this possibility, we generated lysates of well-fed, mixed stage nematodes from either wild type or *uev-1* mutants, and then separated the proteins from these lysates by SDS-PAGE. We used an antibody specific for K63-linked polyubiquitin chains to probe these lysates on Western blots [90]. As controls for specificity, we used SDS-PAGE to separate pure K63-linked and K48-linked tetraubiquitin proteins on the same gels. The anti-K63-linked ubiquitin antibody detected multiple bands of various sizes and quantities (Figure 9B). Interestingly, we found the same pattern of K63-linked ubiquitinated proteins in both wild-type and *uev-1*-mutant lysates. Importantly, the antibody detected K63-linked but not K48-linked tetraubiquitin control proteins, supporting its specificity. Similar results were found in five independent immunoblots. Our results indicate that UEV-1 does not affect the overall levels of K63-linked polyubiquitination in *C. elegans*.

A viable deletion mutant for *C. elegans ubc-13* has recently been identified (Figure S5; [91]). To directly test whether UEV-1 might regulate GLR-1 trafficking by working with UBC-13, we introduced the *P_glr-1_::glr-1::gfp* transgene into *ubc-13* mutants. Similar to *uev-1* mutants, *ubc-13* mutants accumulate GLR-1::GFP in their neuron cell bodies and proximal neurites (Figure 9C-F). In addition, double mutants for *uev-1* and *ubc-13* show quantitatively and qualitatively similar phenotypes compared to both single mutants (Figure 9C-F and data not shown), suggesting that UEV-1 and UBC-13 might work as a heterodimer to regulate GLR-1 trafficking, most likely by mediating K63-linked polyubiquitination. Given that we did not observe changes in global levels of K63-linked polyubiquitinated proteins in *uev-1* mutants, we speculate that UEV-1 controls the ubiquitination of a small number of specific substrates (present at low enough levels to escape detection by our anti-K63 Western analysis), and that one or more of these substrates has a key role in regulating GLR-1 trafficking out of RAB-10-containing endosomes.

## Discussion

We have identified an *in vivo* role for UEV-1 in a metazoan, *C. elegans*, in regulating the trafficking of an AMPA-type glutamate receptor subunit, GLR-1. We find that while UEV-1 is broadly expressed in *C. elegans* cells and tissues, it causes a specific defect in the localization of GLR-1, which can be rescued cell autonomously in GLR-1-expressing interneurons by expression of a *uev-1* cDNA. Our evidence indicates that UEV-1 likely does not function as part of the ERAD pathway, but in determining this we uncovered a strong spatial preference for where GLR-1 accretions form along the ventral cord of *C. elegans* in *uev-1* mutants. We provide evidence that GLR-1 accumulates at RAB-10-containing endosomes in *uev-1* mutants, and that receptors arrive at these endosomes independent of clathrin-mediated endocytosis. UEV-1 homologs found in other species are thought to function by binding to Ubc13, with the resulting heterodimers forming K63-linked ubiquitin chains. We find that whereas UEV-1 can interact with *C. elegans* UBC-13, global levels of K63-linked ubiquitination seem unaffected in *uev-1* mutants. Nevertheless, UBC-13, like UEV-1, is required to regulate GLR-1 trafficking. Our results suggest that UEV-1 and UBC-13 could regulate a small subset of K63-linked ubiquitination events in nematodes, at least one of which is critical in regulating GLR-1 trafficking.

### UEV-1 might work in conjunction with UBC-13 in *C. elegans* to regulate the K63-linked ubiquitination state of one or more substrates

UEV-1 is similar in sequence to other proteins in the highly conserved UEV family, which are similar in sequence and structure to the ubiquitin E2 conjugating enzymes, but lack the catalytic cysteine [31]–[35]. UEV family members have been implicated both in generating K63-linked ubiquitin chains, as well as in the recognition and binding of previously ubiquitinated substrate proteins. For example, UEV-1 is similar to TSG101, itself a homolog of the yeast ESCRT protein Vps23 [33], [85]. ESCRT proteins function to route ubiquitinated receptors from the early endosome to multivesicular bodies and eventually to lysosomes, and Vps23 is a subunit of ESCRT-1, which binds ubiquitinated receptors in the early endosome [36]. Therefore, one possible mechanism of action for UEV-1 in regulating GLR-1 trafficking could be by functioning as part of the ESCRT complex. However, mutations in known ESCRT complex genes do not appear to affect GLR-1 trafficking at a gross level. In addition, UEV-1 does not appear to be localized to endosomes, as would be expected for a Vps23 homolog and ESCRT complex protein. Finally, the overall levels of GLR-1 are not different in *uev-1* mutants compared with wild type, suggesting that the GLR-1 accretions observed in *uev-1* mutants are not simply a result of elevated GLR-1 protein levels, but instead are due to a change in GLR-1 trafficking and subcellular localization.

Another possibility is that UEV-1 forms a heterodimer with UBC-13 [38] to catalyze the formation of K63-linked polyubiquitin chains. In other organisms, K63-linked polyubiquitination is important in several processes, including lysosomal targeting [27], [30], [40], [41], regulating protein inclusions [42], [43], and protein transport [45]. Consistent with this possible function, RNAi screens have uncovered a role for UEV-1 and UBC-13 in regulating polyglutamine inclusions in *C. elegans* [60]. Importantly, we find that a deletion mutation in *ubc-13* that results in an early nonsense codon (and therefore is likely a null; Figure S5) causes the same GLR-1 trafficking defects as observed in *uev-1* mutants, supporting the idea that a UEV-1/UBC-13 heterodimer complex is regulating GLR-1 trafficking. We find that the levels of K63-linked polyubiquitin are not grossly different in *uev-1* mutants compared to the levels found in wild type, suggesting that UEV-1 is not needed for most K63-linked ubiquitination events in nematodes. *C. elegans* does not contain other homologs of Uev1/Mms2 in its genome. However, the ubiquitin conjugating enzyme UBC-1 contains a short N-terminal amino acid stretch that is similar to that of UEV-1, and UBC-1 can interact with UBC-13 in a yeast two-hybrid assay [59], [92]. UBC-13 can also interact with the E3 ubiquitin ligase NHL-1, and together UBC-13, UBC-1, and NHL-1 can form polyubiquitin conjugates *in vitro* [59]. Thus, one possible explanation is that UBC-13 conducts most K63-linked ubiquitination while working with UBC-1 rather than when partnered with UEV-1. We examined GLR-1 localization in null mutants containing deletions for *ubc-1* and *nhl-1*, but did not observe defects (data not shown). We also generated *uev-1 ubc-1* double mutants and found that they were viable and had similar GLR-1 trafficking defects to *uev-1* single mutant animals (data not shown). We speculate that UEV-1 regulates the K63-linked ubiquitination of a small number of substrates, one or more of which is involved in GLR-1 trafficking, although we acknowledge that we cannot rule out the alternative possibility that UEV-1 and UBC-13 regulate GLR-1 trafficking by binding ubiquitinated proteins rather than catalytically modifying their K63 linkages.

Does UEV-1 regulate the polyubiquitination of GLR-1 subunits directly? We found that *uev-1* mutants contain GLR-1 in accretions and possess the same defects in spontaneous reversal frequency even when the four lysines that are normally ubiquitinated on the tail of GLR-1 are mutated to arginine to prevent their ubiquitination. Preventing direct ubiquitination of GLR-1 does not substantially suppress the *uev-1* mutant phenotype. Thus, it seems unlikely that UEV-1 regulates GLR-1 trafficking via direct polyubiquitination of the receptor itself, although we cannot rule out a subtle role for direct ubiquitination of the receptor. Instead, our data suggests that UEV-1 is ubiquitinating additional factors that in turn regulate GLR-1 trafficking. Several E3 ligases regulate GLR-1 trafficking indirectly by ubiquitinating key regulators of trafficking [56], [57]. One of these regulators is the PMK-3/p38 MAPK pathway, which is a downstream target of the E3 ligase RPM-1 in both the command interneurons and the motoneurons [56], [68]. Mutants for *uev-1* have similar defects to *rpm-1* mutants with regard to these different neuron types, and mutations in *pmk-3* suppress the defects in *rpm-1* mutants in both neuron types. However, mutations in *pmk-3* do not suppress the GLR-1 trafficking defects or the motoneuron synaptic differentiation defects in *uev-1* mutants, suggesting that p38 MAPK signaling is not the target of UEV-1 activity.

Interestingly, we detected UEV-1 enrichment in nuclei. K63-linked ubiquitination of histones has been observed in response to DNA damage [93]–[95]. While we have not found any evidence to suggest that UEV-1 functions in a DNA damage response pathway, it remains possible that UEV-1 regulates GLR-1 by affecting changes in transcription in the nucleus. We do not see an effect of *uev-1* on other reporters expressed by the *glr-1* promoter or on *glr-1* mRNA levels, so it is unlikely that UEV-1 regulates *glr-1* transcription; however, we cannot exclude the possibility that UEV-1 regulates the transcription of yet unknown factors that in turn regulate GLR-1 trafficking.

### UEV-1 controls the exit of GLR-1 from early endosomes

A lower frequency of spontaneous reversals in *C. elegans* has previously been shown to correlate with less GLR-1 in the postsynaptic membrane of GLR-1 expressing neurons [47]–[49], [51]. We found that the rate of reversals was significantly lower in *uev-1* mutants than in wild type, suggesting that a large amount of the receptor is in an intracellular compartment. In addition, we found that in *uev-1* mutants there is a significant increase in the colocalization of GLR-1::GFP with mRFP::RAB-10, which is associated with early endosomes, suggesting that UEV-1 regulates the flow of GLR-1 receptors into or out of the early endosome.

We also found that mutations or transgenes designed to suppress clathrin-dependent endocytosis were not able to suppress the internal accumulation of GLR-1::GFP seen in *uev-1* mutants. In addition, we found that the effect of *uev-1* mutations on GLR-1 trafficking is additive when combined with *lin-10* mutations (in which the recycling of clathrin-dependent cargo is impaired), suggesting that these two genes regulate GLR-1 trafficking by different genetic pathways. By contrast, GLR-1 trafficking defects in *rab-10 uev-1* double mutants are not significantly greater than in *uev-1* single mutants, suggesting that RAB-10 and UEV-1 might function in the same genetic pathway. RAB-10 mediates the recycling of clathrin-independent cargo [55]; thus, if UEV-1 and RAB-10 work in the same pathway, this would position UEV-1 as a mediator of GLR-1 recycling in the clathrin-independent endocytosis pathway from endosomes back to synaptic membranes. Consistent with this hypothesis, *uev-1* mutants, like *rab-10* mutants, have a reversal phenotype indicative of reduced GLR-1 synaptic function. It should be noted that *uev-1* null mutants have a stronger GLR-1 accumulation defect than *rab-10* null mutants, suggesting either that UEV-1 is a more critical component than RAB-10 in this recycling pathway, or UEV-1 regulates GLR-1 trafficking by one or more additional mechanisms than just the clathrin-independent recycling pathway.

**Figure 10:**
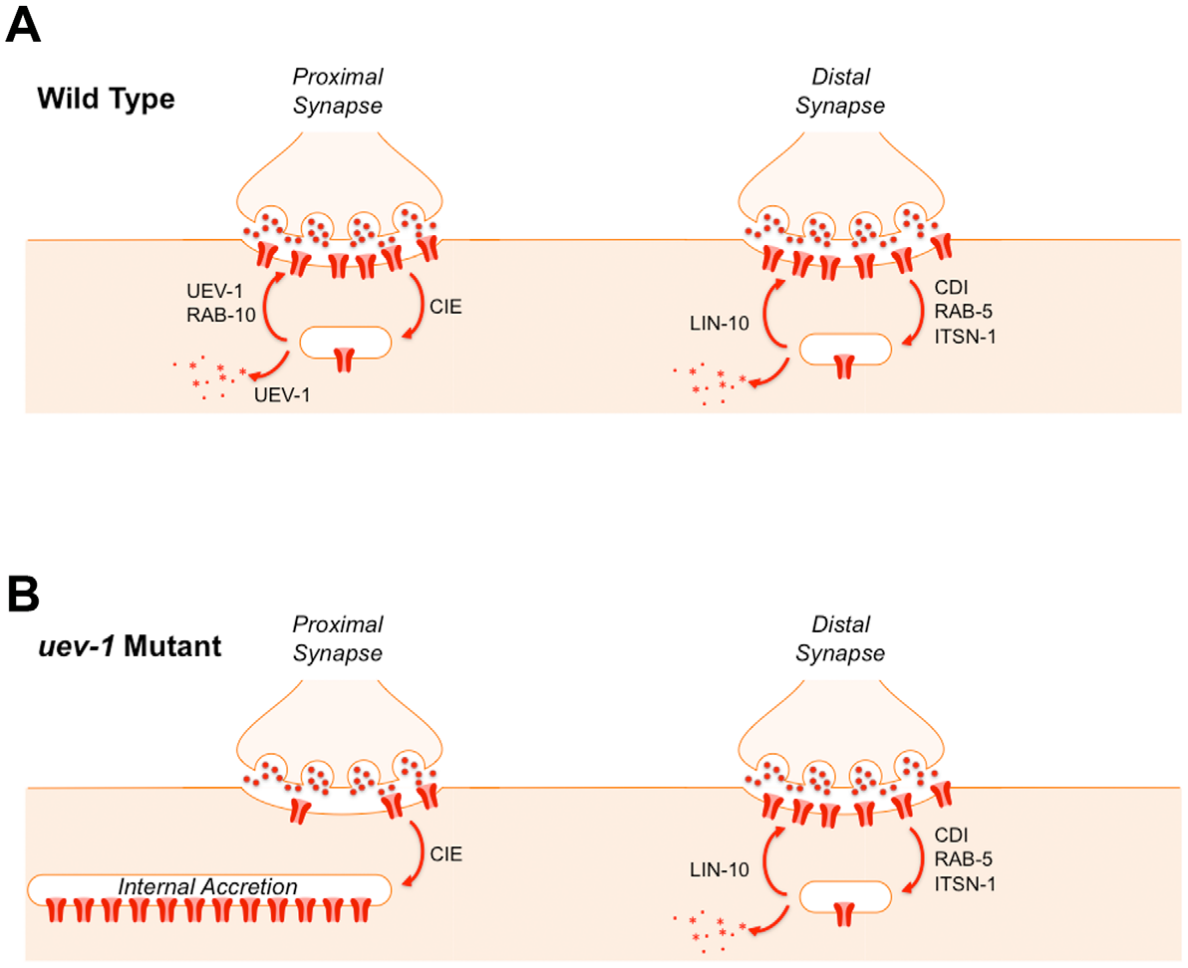
A model for UEV-1 function. Previous results indicate that GLR-1 is endocytosed and recycled by two pathways: a clathrin-dependent pathway (which involves ITSN-1 and RAB-5) that requires LIN-10 for recycling, and a clathrin-independent pathway that requires RAB-10 for recycling. Based on genetic and cell biological data, we suggest that UEV-1 functions in the clathrin independent pathway to move receptors out of endosomes for recycling and for turnover. (A) Cartoon illustrating both pathways at different synapses along the ventral cord of wild-type animals. GLR-1 receptors (red) are being endocytosed and recycled by either the clathrin-independent endocytosis pathway (CIE) and RAB-10 (the synapse on the left) or the clathrin-dependent endocytosis pathway (CDE) and LIN-10 (the synapse on the right). Our results suggest that the more proximal synapses favor the clathrin-independent, UEV-1 dependent pathway. (B) Cartoon illustrating that the clathrin-independent pathway is disrupted in *uev-1* mutants, resulting in the internalization of GLR-1 receptors into accretions due to their failure to exit endosomal compartments.

This leads us to entertain a model in which UEV-1 functions in GLR-1-expressing interneurons to facilitate the exit of GLR-1 from early endosomes (Figure 10A). A fraction of GLR-1 receptors is normally endocytosed by a clathrin-independent pathway and sent to RAB-10-containing early endosomes [55]. Subsequently, GLR-1 receptors exit these endosomes and are either recycled to the synapse or sent to lysosomes for turnover. In the absence of UEV-1 function, a fraction of GLR-1 receptors become endocytosed into these endosomes, but cannot exit them, resulting in a net build up of receptor in the neurons while simultaneously depleting the synaptic surface of receptor and resulting in decreased GLR-1 signaling (Figure 10B). Interestingly, we observed a spatial bias in the localization defects in *uev-1* mutants, suggesting that these endosomes might lie more proximal along neurites. This finding implies that neurons can control the spatial localization of glutamate receptors along neurites by regulating the balance of the different endocytosis pathways that regulate the receptors. Our model is consistent with work showing a role for K63-linked polyubiquitination in the trafficking of renal aquaporin-2 water channels [27], the epidermal growth factor receptor [41], Gap1 permease [30], and other proteins [40], with the novel feature that the GLR-1 receptor itself is not the likely K63-linked ubiquitination target. Finally, this model raises the possibility that UEV proteins are important for regulating AMPAR function, which could have implications for synaptic plasticity and neurodegeneration in other organisms, including mammals. Most studies of UEV protein function to date have been in single celled organisms or have been in tissue culture. Our study indicates that the *in vivo* roles of UEV proteins in metazoans are likely to be even broader than originally expected.

## Materials and Methods

### Strains

Animals were grown at 20°C on standard NGM plates seeded with OP50 *E. coli*. Some strains were provided by the Caenorhabditis Genetics Center. Most strains were backcrossed multiple times to our laboratory N2 strain to minimize other genetic variation. The following strains were used: *alx-1(gk338), ire-1(ok799), itsn-1(ok268), juIs1[P_unc-25_::SNB-1::GFP], lin-10(e1439), nhl-1(gk15), nuIs108[GLR-1(4KR)::GFP], nuIs24[P_glr-1_::GLR-1::GFP], nuIs25[P_glr-1_::GLR-1::GFP], nuIs68[P_glr-1_::UNC-43::GFP], nuIs89[P_glr-1_::MUb], odEx[F39B2 cosmid, rol-6dm], odEx[P_glr-1_::mCherry::UEV-1, rol-6dm], odEx[P_glr-1_::SYN-13::RFP], odEx[P_glr-1_::UEV-1(cDNA), rol-6dm], odEx[P_glr-1_::UEV-1(F8A cDNA), rol-6dm], odEx[P_glr-1_::UEV-1(F8A mini-gene)], odEx[P_glr-1_::UEV-1(mini-gene)], odEx[P_glr-1_::UEV-1(mini-gene)], odEx[P_glr-1_::UEV-1(genomic)], odEx[P_glr-1_::UEV-1::GFP], odEx[Rab-5(S23N)], odEx[rfp::rme-1], odIs1[P_glr-1_::SNB-1::GFP], odIs22[P_glr-1_::LIN-10::GFP], odIs42[P_glr-1_::RAB-10::RFP], pdr-1(tm395), pdr-1(tm598), pmk-3(ok169), stam-1(ok406), rab-10(q373), rpm-1(js317), trf-1(nr2014), ubc-1(gk14), uev-1(od10), uev-1(ok2610), ubc-13(tm3546),* and the CB4856 Hawaiian strain.

### Transgenes and Germline Transformation

Transgenic strains generated in this study were isolated after microinjecting various plasmids (5–50 ng/ml) using *rol-6dm* (a gift from C. Mello, UMass), *ttx-3::rfp* (a gift from O. Hobert, Columbia Univ.), or *lin-15(+)* (a gift from J. Mendel, CalTech) as a marker. Plasmids containing the *glr-1* or *uev-1* promoters, followed either by a *uev-1* minigene (wild type or F8A mutant), *uev-1* cDNA (wild type or F8A mutant), *uev-1* genomic, or *mCherry::uev-1*, were generated using standard techniques. All resulting transgenes were introduced into the germline and followed as extrachromosomal arrays.

### Isolation and mapping of *uev-1(od10)*

P0 *nuIs25* nematodes were EMS mutagenized using standard procedures. F2 animals from individual plates were sampled (n = 30–50) by mounting on 2% agarose pads containing levamisole. Animals were scored by fluorescence microscopy for defects in GLR-1::GFP localization. Mutants were recovered either directly from the slide or by isolating siblings from the parental F1 plate. Mutants were further characterized after 4 rounds of backcrossing.

The *od10* mutation was closely linked to *unc-54* (map position +27.4), and was three-factor mapped between SNP *pkP1071* (map position +23.4) and SNP *pkP1072* (map position +28.48) on the right arm of LGI by crossing marked *uev-1* strains to the polymorphic strain CB4856. We detected 20 recombination events between *pkP1071* and *od10*, and 2 recombination events between *od10* and *pkP1072*, suggesting that *od10* mapped to around +27.0. We injected genomic cosmids covering ∼200 kb around the *unc-54* locus, and found that cosmid F39B2 rescued the *uev-1* mutant phenotype. This cosmid contains 12 genes, including *uev-1*. We sequenced several candidate genes from *od10* mutant genomic DNA, and identified a nonsense mutation in the *uev-1* gene. Expression of a *uev-1* minigene containing *uev-1* coding sequences and 2 kilobases of promoter was also sufficient to rescue the *uev-1* mutant phenotype.

### Fluorescence Microscopy

GFP- and RFP-tagged fluorescent proteins were visualized in nematodes by mounting larvae on 2% agarose pads with levamisole. Fluorescent images were observed using a Zeiss Axioplan II. A 100X (N.A. = 1.4) PlanApo objective was used to detect GFP and RFP signals. Imaging was done with an ORCA charge-coupled device (CCD) camera (Hamamatsu, Bridgewater, NJ) using IPLab software (Scanalytics, Inc, Fairfax, VA) or iVision v4.0.11 (Biovision Technologies, Exton, PA) software. Exposure times were chosen to fill the 12-bit dynamic range without saturation. In most cases, maximum intensity projections of z-series stacks were obtained and out-of-focus light was removed with a constrained iterative deconvolution algorithm (Vaytek). For most images, we captured the ventral cord neurites in the retrovesicular ganglion region surrounding the RIG and AVG cell bodies.

The quantification of ventral nerve cord fluorescent objects (i.e., puncta and accretions) was done using ImageJ [96] to automatically threshold the images and then determine the outlines of fluorescent objects in ventral cord neurites. ImageJ was used to quantify both the shape and the size of all individual fluorescent objects along the ventral cord. This allowed us to distinguish between the small GLR-1::GFP puncta in wild-type animals and the large, aberrant accretions (which have an elongated shape not observed in wild type) in *uev-1* and other mutants. Object size was measured as the maximum diameter for each outlined cluster. Object number was calculated by counting the average number of clusters per 100 microns of dendrite length.

To quantify the fluorescent intensities of individual GLR-1::GFP puncta and accretions along the ventral cord neurites, a 20X objective was used to capture images of animals, and a median filter was used to subtract away the background nematode and coverslip autofluorescence. Line profiles were drawn along the length of the ventral cord starting from the anterior end of the retrovesicular ganglion and moving posterior for approximately 250 microns to show the fluorescent intensity peaks for individual puncta and accretions. For quantification of ventral cord fluorescence, ImageJ was used to measure the integrated fluorescent density (the sum of all detectable pixel intensities along a given ventral cord process) normalized to the length of that process.

The quantification of PVC cell body fluorescence was done using ImageJ to measure the integrated fluorescent density (the sum of all detectable pixel intensities per cell body) for each neuron. For the quantification of GLR-1::GFP and mRFP::RAB-10 colocalization, we fixed animals with ice cold 1% paraformaldehyde in PBS for 10 minutes and imaged them using a previously published protocol [56], [76]. Images for neuronal cell bodies were taken using a Carl Zeiss confocal microscope equipped with the BD CARV IITM Confocal Imager and a Carl Zeiss 100X Plan-Apochroma objective (N.A. = 1.4).

For quantitative colocalization analysis, all image manipulations were performed with iVision v4.0.11 (Biovision Technologies, Exton, PA) software using the FCV colocalization function. We applied an empirically derived threshold to all images for both the GLR-1::GFP channel and the mRFP::RAB-10 channel to eliminate background coverslip fluorescence and other noise (typically, 5% of pixels for each channel). The fluorescent intensity values for both the GLR-1::GFP and mRFP::RAB-10 channels were then scatter plotted for each pixel. Pixels with similar intensity values for both channels (within an empirically established tolerance factor) were counted as colocalized. To acquire the fraction of GLR-1::GFP colocalized with mRFP::RAB-10, the number of colocalized pixels was normalized to the number of GLR-1::GFP pixels under threshold. To maximize our resolving power while observing the relatively small *C. elegans* neuron cell bodies, we restricted our analysis to a single focal plane taken through the middle of each cell body. As an alternative approach, we also analyzed the same images using a confined displacement algorithm run in ImageJ as previously described [79]. Manders colocalization coefficients were determined for the real data and for the randomized image, and then compared statistically to obtain *p* values.

### Behavioral Assays

The reversal frequency of individual animals was assayed as previously described, but with some modifications [51]. Single young adult hermaphrodites were placed on NGM plates in the absence of food. The animals were allowed to adjust to the plates for 5 minutes, and the number of spontaneous reversals for each animal was counted over a 5-minute period. Twenty animals were tested for each genotype, and the reported scores reflect the mean number of reversals per minute.

### GST Pull Downs

Complete sequences for *ubc-13* were introduced into the GST expression vector pGEX-GW (a gift from Barth Grant), and either GST alone or GST::UBC-13 were expressed in *E. coli* strain BL21 and purified using glutathione-Sepharose as described previously [97]. Complete sequences for *uev-1* or *uev-1* mutated F8A were introduced into the pcDNA3.1-GFP expression vector (a gift from Barth Grant), and transfected in COS7 cells for 48 hours. Cell were harvested and lysed in TEEN (20 mM Tris, 1 mM EDTA, 1mM EGTA, 100 mM NaCl) plus 1 mM PMSF, and Triton X-100 was added to 1% for 1 hour at 4°C after needle aspiration. For each experiment, 12 micrograms of GST protein was added to 400 microliters of solubilized supernatant, incubated for 45–60 minutes at 4°C, and washed several times in TEEN plus 0.2% Triton X-100. Proteins were eluted into loading buffer and separated by SDS-PAGE for Western blot analysis using anti-GFP antibodies.

### Yeast Two-hybrid Interactions

Yeast two-hybrid experiments were performed by placing *ubc-13* and *uev-1* cDNA sequences into the pEG202 bait vector and pJG4-5 prey vector. The resulting plasmids were cotransformed, along with the reporter plasmid pSH18-34, into yeast strain EGY48, and transformed yeast were recovered on –His –Trp –Ura dropout plates. Resulting colonies were diluted in series on –Leu –His –Trp dropout plates to test for interactions based on growth.

### K63-linked Anti-ubiquitin Western Blotting

Western blots with Apu3.A8 were performed essentially as described previously with modifications [90]. Nematodes were dounce homogenized, needle aspirated, and sonicated in 20 mM Tris (pH 7.4), 1 mM EGTA, 10% glycerol, 135 mM NaCl, 1.5 mM MgCl_2_, 1% Triton X-100, 6M urea, 2 mM N-ethylmaleimide, 25 micromolar MG132, and a protease inhibitor cocktail (Roche). Total protein was quantified by Bradford assay, and then SDS was added to a final concentration of 1%. The lysates was centrifuged at 13,000xg to remove insoluble material, and equal quantities of extracted proteins were separated on SDS-PAGE gels. After electrophoresis, resolved proteins were transferred to PVDF membranes (Immobilon-P) and blocked with 2% BSA in PBS with Triton X-100. Membranes were incubated with Apu3.A8 anti-K63-linked ubiquitin antibodies (Millipore) at 1:300 for 1 hour, or C4 monoclonal anti-actin antibodies (MPBiomedic) at 1∶1000 overnight at 4°C. After washing, HRP-conjugated secondary antibody was applied at 1∶2000 for 1 hour at room temperature. Immunoreactive bands were visualized using the enhanced chemiluminescence (ECL) system (GE Healthcare).

### GLR-1::GFP Western Blotting

Lysates were prepared from adult worms using a Wheaton DuraGrind stainless steel dounce homogenizer and buffer A (50 mM Hepes pH 7.7, 50 mM potassium acetate, 2 mM magnesium, 1mM EDTA, 250 mM sucrose), a protease inhibitor cocktail (Roche), and 10 mM N-ethylmaleimide. Membranes were isolated from clarified lysates by ultracentrifugation, and then suspended in buffer A plus β-mercaptoethanol, SDS, and DTT. Proteins were separated from membrane lysates by SDS-PAGE, and GLR-1::GFP or actin were simultaneously detected by Western blotting using a combination of anti-GFP antibodies (GeneTex Inc.) and anti-actin antibodies (MP Biomedicals). Secondary antibodies conjugated to infrared-emitting tags were used to detect both primaries on the blot using an Odyssey LI-COR. Quantitation was performed by comparing samples to a standard curve generated by diluting samples from wild-type lysates.

### Real-time PCR

Total RNA from adult worms was prepared using Trizol (Invitrogen). First strand reverse transcription was performed using iScript (Biorad) as per the manufacturer's instructions. Real-time PCR reactions were performed using iQ SYBR Green (Biorad) and a BioRad real-time PCR cycler as previously described [63]. Primer sets detected either *glr-1* mRNA or mRNA for *dlg-1*, a control transcript of an adherens junction protein [98]. Primers spanned intron junctions so as not to detect genomic DNA, and no product was detected in the absence of reverse transcription. Standard curves were generated and analyzed in triplicate by dilution. Concentrations were derived by comparing Ct values of samples to standard curves. For each genotype, mean *glr-1* expression was normalized to that of *dlg-1.*

## Supporting Information

Figure S1 The uev-1 transcription unit. Genomic sequences in and around the uev-1 transcription unit, from the start of transcription until the final nucleotide present in the mRNA, are shown. Capital letters highlighted in gray indicate coding sequences within exons. Numbers are based on nucleotides starting from the ATG and containing only exonic sequences. The horizontal bar indicates sequences missing in the ok2610 deletion. The nonsense mutation in od10 is also indicated.
(2.68 MB TIF)

Figure S2 Overview of GLR-1-Expressing Neurons. Top panel diagram indicates the position of the pharynx (green), command interneuron cell bodies (red circles, only left side shown for clarity), RIG cell bodies (blue circles), AVG cell body (purple circle), and the various neurite projections into the nerve ring and along the ventral cord in the head region. The PVC command neuron cell body, which is located in the tail, is not show; however, the PVC neurite would belong to the bundle of fibers along the ventral cord and projecting into the nerve ring as indicated in red. Note that the RIG neurites do not enter the ventral cord until the most anterior portion of the lateral ganglion; thus, no RIG synapses are included in our analysis [100]. Also note that AVG makes only a single synapse in the retrovesicular ganglion region of the ventral cord; thus, the contribution of AVG to our analysis is minimal [100]. Bottom panel diagram indicates the position of cell bodies and neurites from a dorsal view. The circular nerve ring has been flattened out to lie in the same plane as the ventral cord so that the posterior face of the nerve ring and the dorsal face of the ventral cord are directed out of the page. The lateral ganglion and retrovesicular ganglion regions are indicated by brackets.
(3.25 MB TIF)

Figure S3 GLR-1 mRNA and protein levels do not vary significantly in uev-1 mutants compared to wild type. (A) The levels of glr-1 mRNA relative to dlg-1 (a control adherens junction protein) mRNA as detected by qRT-PCR are shown for the indicated genotypes. N = 5 trials. (B) The levels of GLR-1::GFP protein relative to actin protein as detected by quantitative Western blotting are shown. (C) A sample Western blot for GLR-1::GFP (top) and actin (bottom) is shown. N = 3 trials.
(1.00 MB TIF)

Figure S4 GLR-1 colocalization with RAB-10 and SYN-13 as analyzed by a confinement displacement algorithm. (A,D) GLR-1::GFP fluorescence and (B,E) SYN-13::RFP fluorescence from (A-C) wild type or (D-F) uev-1 mutants. (C,F) Merged images. The mean Manders colocalization coefficient is shown for (G) GLR-1 that colocalizes with RAB-10, (H) RAB-10 that colocalizes with GLR-1, (I) GLR-1 that colocalizes with SYN-13, and (J) SYN-13 that colocalizes with GLR-1 for the indicated genotypes. Gray bars indicate the coefficients determined from the original fluorescent images. Red bars indicate the coefficients determined from the same images randomized by a confinement displacement algorithm, thus measuring the probability that the correlation coefficients for colocalization are occurring my random chance within the small confined space of these cells [79]. *P<0.05, **P<0.01, ***P<0.001 by ANOVA with the indicated Bonferonni comparisons. N = 15-22 animals for each genotype.
(9.38 MB TIF)

Figure S5 The ubc-13 transcription unit. (Top) The intron/exon structure of ubc-13 based on sequenced cDNAs is shown in the top panel. Gray boxes indicate exonic coding sequences. The arrow indicates the start of transcription. The purple line indicates the sequences that are removed by the tm3546 deletion. (Bottom) Genomic sequences in and around the ubc-13 transcription unit, from the start of translation until the final stop codon present in the mRNA, are shown. Capital letters highlighted in gray indicate coding sequences within exons. Numbers are based on amino acids starting from the ATG and containing only exonic sequences. The horizontal bar indicates sequences missing in the tm3546 deletion. The deletion removes the 5' splice site and results in an immediate nonsense codon following the deletion breakpoint; thus, only 88 amino acids of the protein at most are produced.
(1.08 MB TIF)

Figure S6 Clathrin-dependent and clathrin-independent pathways regulate GLR-1 trafficking. For each genotype, a cartoon is shown of two synapses along the ventral nerve cord bundle, with predictions based on our hypothesis for UEV-1 function. GLR-1 receptors (red) are being endocytosed and recycled by either the clathrin-independent endocytosis pathway (CIE) and RAB-10 (the synapse on the left) or the clathrin-dependent endocytosis pathway (CDE) and LIN-10 (the synapse on the right). (A) Trafficking in wild-type animals, based on our model and previously published results [55], [56]. (B) In lin-10 mutants, GLR-1 is endocytosed by CDE, including RAB-5 and ITSN-1; however, receptors are not recycled and accumulate in internal endosomes. (C) In uev-1 mutants, rab-10 mutants, or uev-1 rab-10 double mutants, our findings suggest that GLR-1 is endocytosed by CIE; however, receptors are not recycled and accumulate in internal endosomes. (D) Since UEV-1 and LIN-10 are expected to regulate GLR-1 by these two separate pathways, we would expect an increase in the amount of internalized GLR-1 in uev-1 lin-10 double mutants. (E) Mutations that reduce CDE (e.g., itsn-1) should not block the accumulation of GLR-1 in intracellular compartments in uev-1 mutants, but (F) they do block the internalization of GLR-1 in lin-10 mutants [55].
(6.57 MB TIF)
