# Supplementary figures and images for: UEV-1 Is an Ubiquitin-Conjugating Enzyme Variant That Regulates Glutamate Receptor Trafficking in C. elegans Neurons
Source: PLoS One. 2010 Dec 13;5(12):e14291. doi: 10.1371/journal.pone.0014291 (PMC3001443; doi:10.1371/journal.pone.0014291)

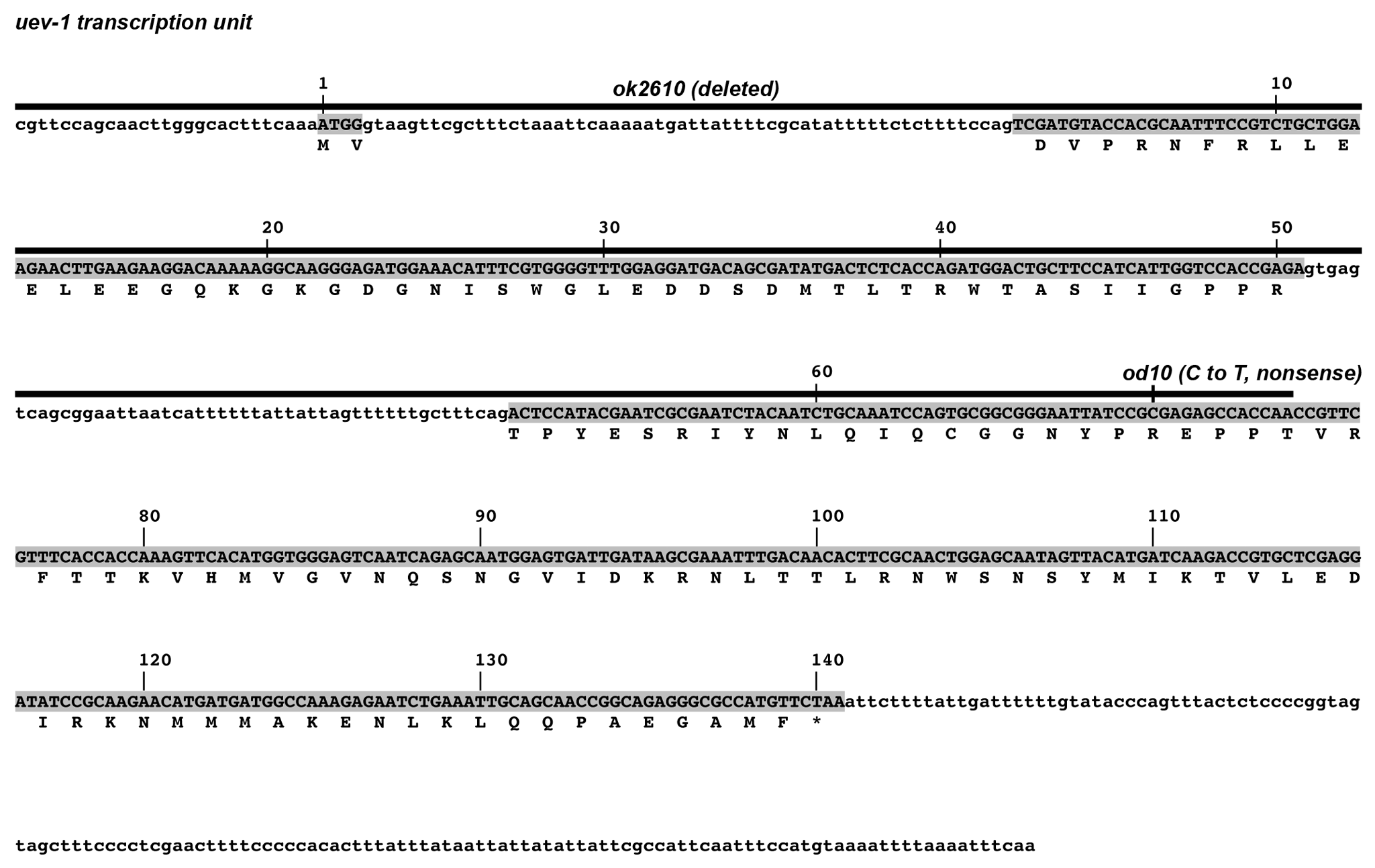

Supplement: Figure S1 — The uev-1 transcription unit. Genomic sequences in and around the uev-1 transcription unit, from the start of transcription until the final nucleotide present in the mRNA, are shown. Capital letters highlighted in gray indicate coding sequences within exons. Numbers are based on nucleotides starting from the ATG and containing only exonic sequences. The horizontal bar indicates sequences missing in the ok2610 deletion. The nonsense mutation in od10 is also indicated. (2.68 MB TIF) [file pone.0014291.s001.tif]

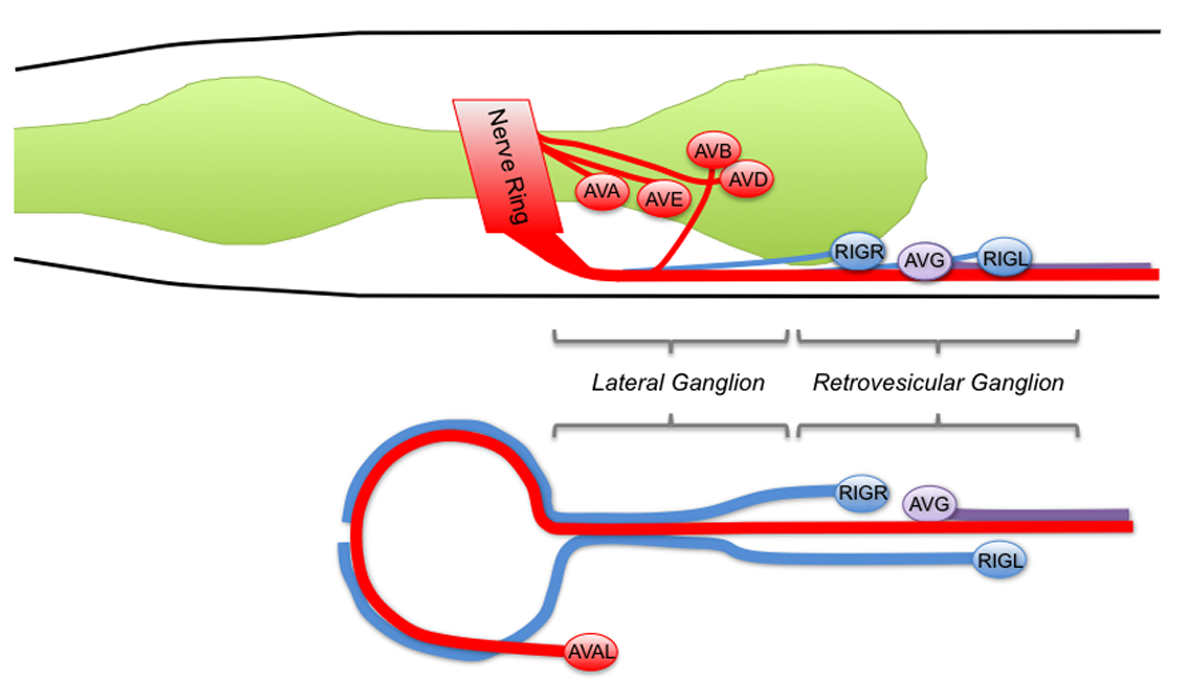

Supplement: Figure S2 — Overview of GLR-1-Expressing Neurons. Top panel diagram indicates the position of the pharynx (green), command interneuron cell bodies (red circles, only left side shown for clarity), RIG cell bodies (blue circles), AVG cell body (purple circle), and the various neurite projections into the nerve ring and along the ventral cord in the head region. The PVC command neuron cell body, which is located in the tail, is not show; however, the PVC neurite would belong to the bundle of fibers along the ventral cord and projecting into the nerve ring as indicated in red. Note that the RIG neurites do not enter the ventral cord until the most anterior portion of the lateral ganglion; thus, no RIG synapses are included in our analysis [100]. Also note that AVG makes only a single synapse in the retrovesicular ganglion region of the ventral cord; thus, the contribution of AVG to our analysis is minimal [100]. Bottom panel diagram indicates the position of cell bodies and neurites from a dorsal view. The circular nerve ring has been flattened out to lie in the same plane as the ventral cord so that the posterior face of the nerve ring and the dorsal face of the ventral cord are directed out of the page. The lateral ganglion and retrovesicular ganglion regions are indicated by brackets. (3.25 MB TIF) [file pone.0014291.s002.tif]

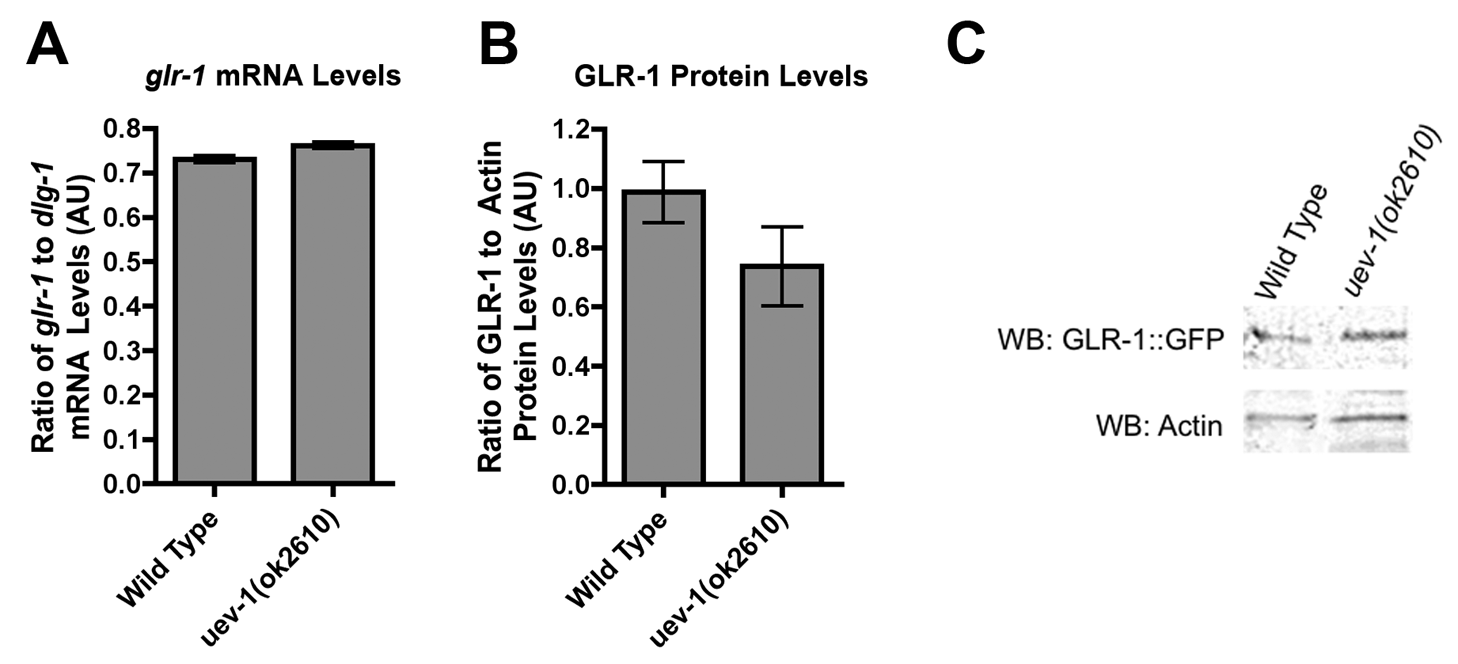

Supplement: Figure S3 — GLR-1 mRNA and protein levels do not vary significantly in uev-1 mutants compared to wild type. (A) The levels of glr-1 mRNA relative to dlg-1 (a control adherens junction protein) mRNA as detected by qRT-PCR are shown for the indicated genotypes. N = 5 trials. (B) The levels of GLR-1::GFP protein relative to actin protein as detected by quantitative Western blotting are shown. (C) A sample Western blot for GLR-1::GFP (top) and actin (bottom) is shown. N = 3 trials. (1.00 MB TIF) [file pone.0014291.s003.tif]

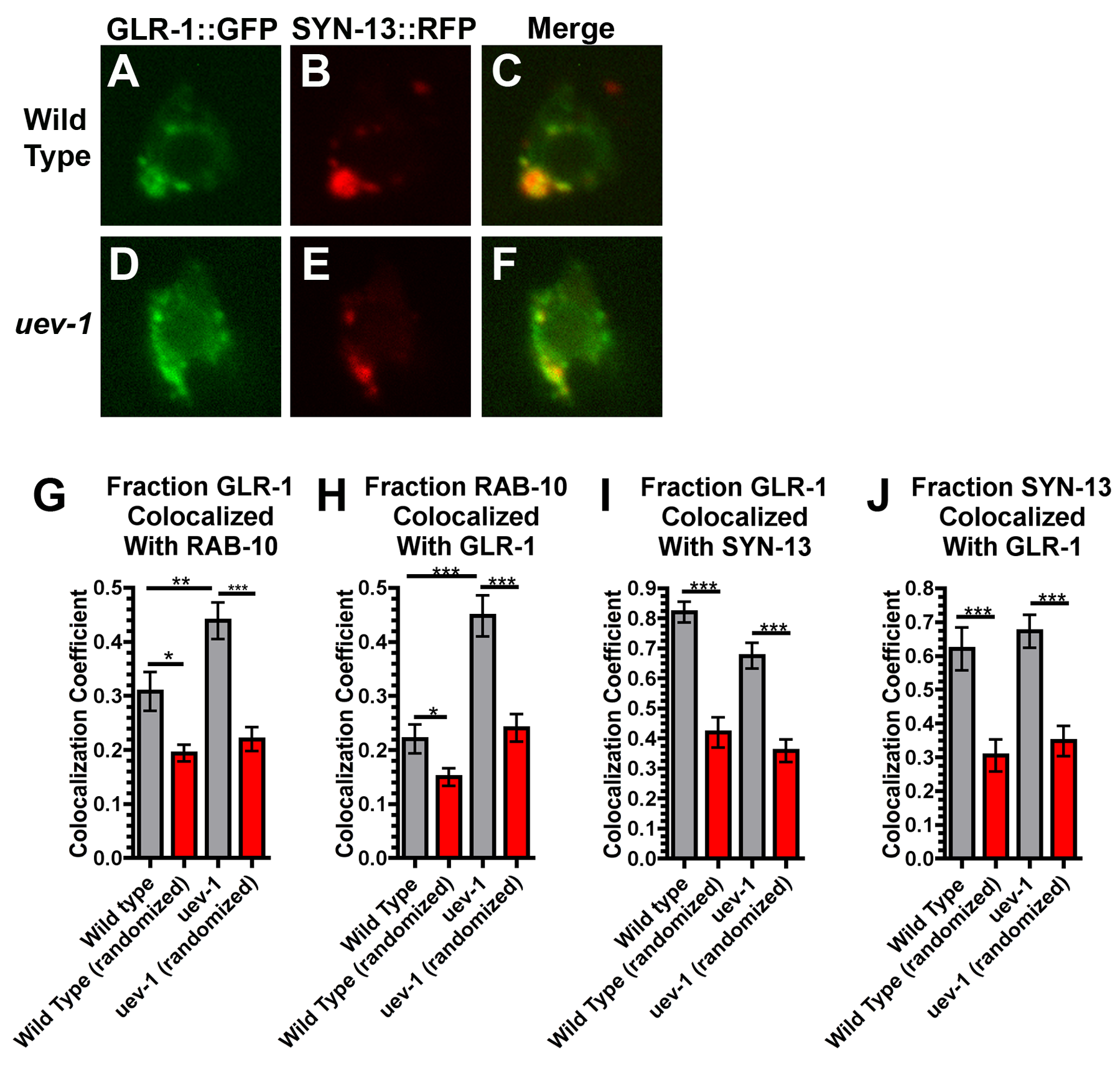

Supplement: Figure S4 — GLR-1 colocalization with RAB-10 and SYN-13 as analyzed by a confinement displacement algorithm. (A,D) GLR-1::GFP fluorescence and (B,E) SYN-13::RFP fluorescence from (A-C) wild type or (D-F) uev-1 mutants. (C,F) Merged images. The mean Manders colocalization coefficient is shown for (G) GLR-1 that colocalizes with RAB-10, (H) RAB-10 that colocalizes with GLR-1, (I) GLR-1 that colocalizes with SYN-13, and (J) SYN-13 that colocalizes with GLR-1 for the indicated genotypes. Gray bars indicate the coefficients determined from the original fluorescent images. Red bars indicate the coefficients determined from the same images randomized by a confinement displacement algorithm, thus measuring the probability that the correlation coefficients for colocalization are occurring my random chance within the small confined space of these cells [79]. *P<0.05, **P<0.01, ***P<0.001 by ANOVA with the indicated Bonferonni comparisons. N = 15-22 animals for each genotype. (9.38 MB TIF) [file pone.0014291.s004.tif]

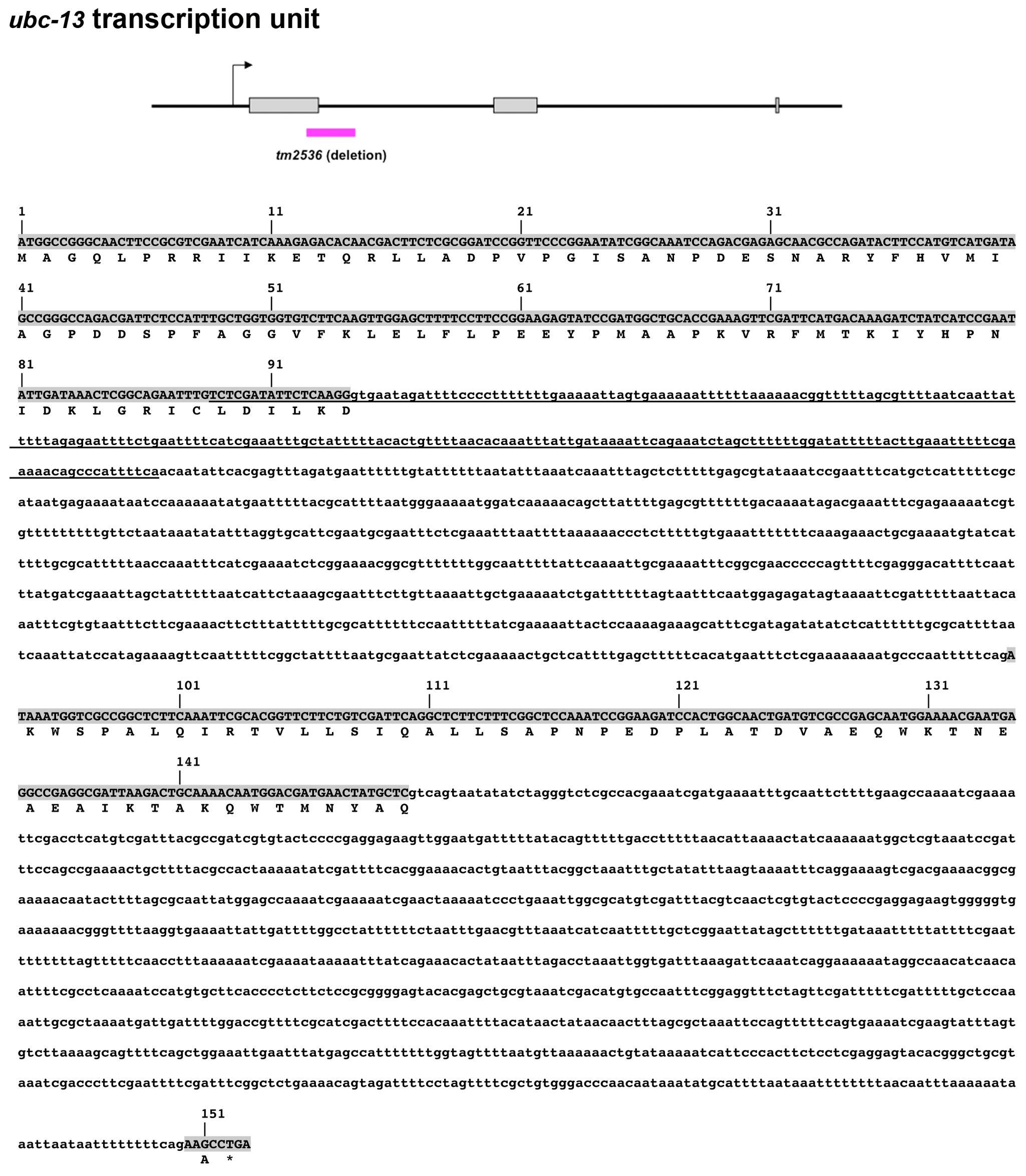

Supplement: Figure S5 — The ubc-13 transcription unit. (Top) The intron/exon structure of ubc-13 based on sequenced cDNAs is shown in the top panel. Gray boxes indicate exonic coding sequences. The arrow indicates the start of transcription. The purple line indicates the sequences that are removed by the tm3546 deletion. (Bottom) Genomic sequences in and around the ubc-13 transcription unit, from the start of translation until the final stop codon present in the mRNA, are shown. Capital letters highlighted in gray indicate coding sequences within exons. Numbers are based on amino acids starting from the ATG and containing only exonic sequences. The horizontal bar indicates sequences missing in the tm3546 deletion. The deletion removes the 5' splice site and results in an immediate nonsense codon following the deletion breakpoint; thus, only 88 amino acids of the protein at most are produced. (1.08 MB TIF) [file pone.0014291.s005.tif]

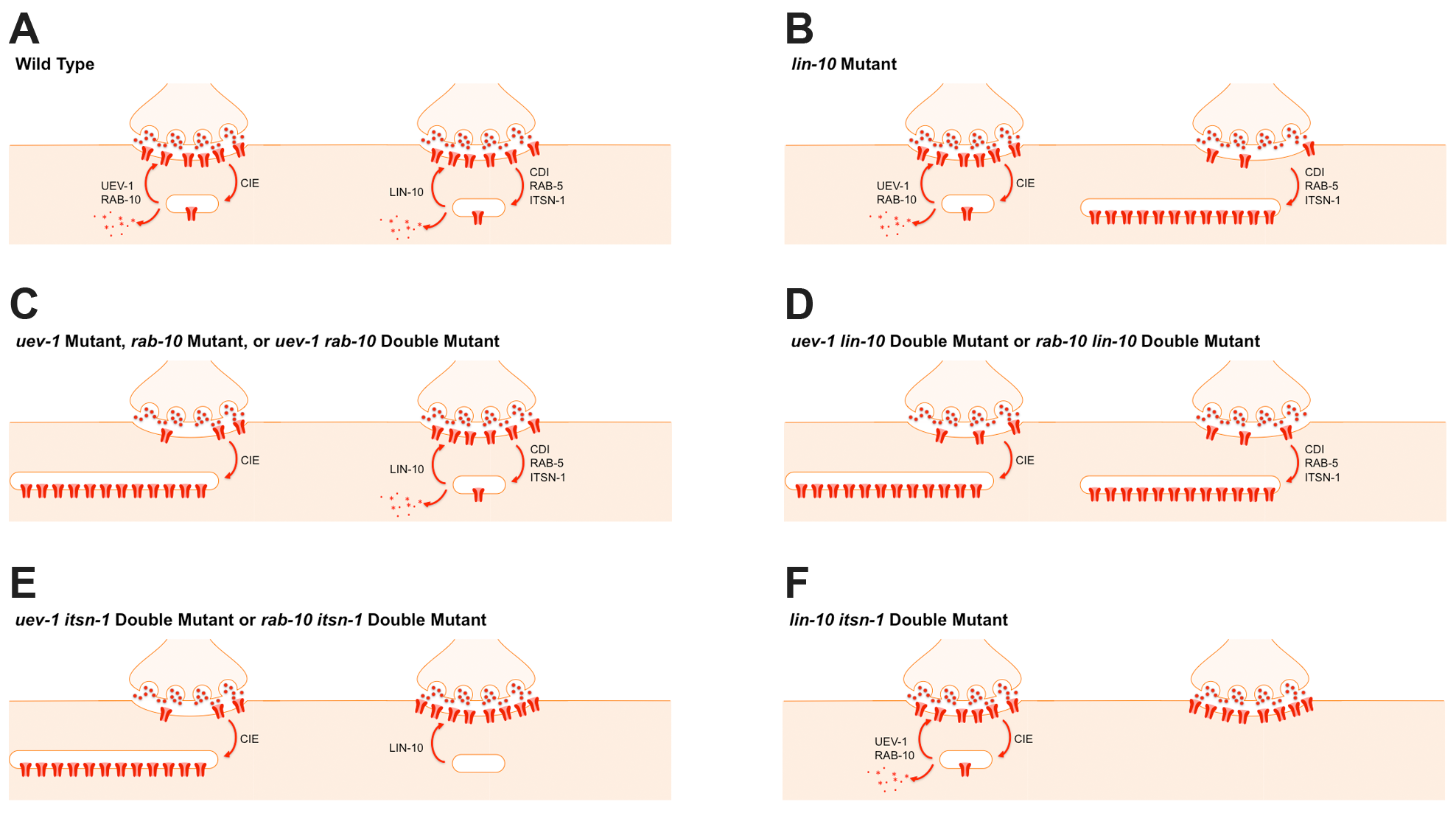

Supplement: Figure S6 — Clathrin-dependent and clathrin-independent pathways regulate GLR-1 trafficking. For each genotype, a cartoon is shown of two synapses along the ventral nerve cord bundle, with predictions based on our hypothesis for UEV-1 function. GLR-1 receptors (red) are being endocytosed and recycled by either the clathrin-independent endocytosis pathway (CIE) and RAB-10 (the synapse on the left) or the clathrin-dependent endocytosis pathway (CDE) and LIN-10 (the synapse on the right). (A) Trafficking in wild-type animals, based on our model and previously published results [55], [56]. (B) In lin-10 mutants, GLR-1 is endocytosed by CDE, including RAB-5 and ITSN-1; however, receptors are not recycled and accumulate in internal endosomes. (C) In uev-1 mutants, rab-10 mutants, or uev-1 rab-10 double mutants, our findings suggest that GLR-1 is endocytosed by CIE; however, receptors are not recycled and accumulate in internal endosomes. (D) Since UEV-1 and LIN-10 are expected to regulate GLR-1 by these two separate pathways, we would expect an increase in the amount of internalized GLR-1 in uev-1 lin-10 double mutants. (E) Mutations that reduce CDE (e.g., itsn-1) should not block the accumulation of GLR-1 in intracellular compartments in uev-1 mutants, but (F) they do block the internalization of GLR-1 in lin-10 mutants [55]. (6.57 MB TIF) [file pone.0014291.s006.tif]
